# Differential CXCR4 expression on hematopoietic progenitor cells versus stem cells directs homing and engraftment

**DOI:** 10.1172/jci.insight.151847

**Published:** 2022-05-09

**Authors:** Sydney Felker, Archana Shrestha, Jeff Bailey, Devin M Pillis, Dylan Siniard, Punam Malik

**Affiliations:** 1 Immunology Graduate Program, Cincinnati Children’s Hospital Medical Center (CCHMC) and the University of Cincinnati College of Medicine, Cincinnati, Ohio, USA.; 2 Division of Experimental Hematology and Cancer Biology and; 3 Division of Hematology, CCHMC, Cincinnati, Ohio, USA.

**Keywords:** Stem cells, Transplantation, Gene therapy, Hematopoietic stem cells, Stem cell transplantation

## Abstract

Gene therapy involves a substantial loss of hematopoietic stem and progenitor cells (HSPC) during processing and homing. Intra-BM (i.b.m.) transplantation can reduce homing losses, but prior studies have not yielded promising results. We studied the mechanisms involved in homing and engraftment of i.b.m. transplanted and i.v. transplanted genetically modified (GM) human HSPC. We found that i.b.m. HSPC transplantation improved engraftment of hematopoietic progenitor cells (HPC) but not of long-term repopulating hematopoietic stem cells (HSC). Mechanistically, HPC expressed higher functional levels of CXCR4 than HSC, conferring them a retention and homing advantage when transplanted i.b.m. Removing HPC and transplanting an HSC-enriched population i.b.m. significantly increased long-term engraftment over i.v. transplantation. Transient upregulation of CXCR4 on GM HSC-enriched cells, using a noncytotoxic portion of viral protein R (VPR) fused to CXCR4 delivered as a protein in lentiviral particles, resulted in higher homing and long-term engraftment of GM HSC transplanted either i.v. or i.b.m. compared with standard i.v. transplants. Overall, we show a mechanism for why i.b.m. transplants do not significantly improve long-term engraftment over i.v. transplants. I.b.m. transplantation becomes relevant when an HSC-enriched population is delivered. Alternatively, CXCR4 expression on HSC, when transiently increased using a protein delivery method, improves homing and engraftment specifically of GM HSC.

## Introduction

Transplantation of hematopoietic stem cells (HSC) is a lifesaving procedure used to treat a variety of blood disorders and cancers. Gene therapy is a variant of autologous HSC transplantation in which a person’s own HSC are reinfused following gene correction, allowing for the avoidance of the risk of graft-versus-host disease or the limitations of a matched donor. Gene therapy is currently most commonly performed using viral vectors (lentiviral [LV] or γ-retroviral vectors), although gene editing is emerging as a future alternative. Gene therapy involves: (a) harvest and enrichment of autologous CD34^+^ hematopoietic stem and progenitor cells (HSPC), (b) their ex vivo culture for genetic manipulation, (c) chemotherapeutic conditioning of the patient (to open the BM HSC niche), and (d) i.v. infusion of the genetically modified (GM) HSPC. The rare HSC (1%–2%) within the CD34^+^ HSPC population home and engraft the BM HSC niche and result in long-term engraftment and GM multilineage blood cell progeny. The success of genetic therapies is dependent upon the ability to achieve sufficient levels of engraftment of GM HSC by maintaining their “stemness” (self-renewal and differentiation capacity) while also achieving high levels of gene transfer. However, a substantial loss of HSPC occurs from collection through transplant. As a result, a much higher HSPC dose is collected, genetically manipulated, and infused than is necessary for standard (non–gene therapy) autologous transplants. Obligate losses include those that occur during HSPC enrichment from harvested BM or peripheral blood apheresis product and from formulation and pretransplant release testing. Losses of HSC that can be reduced include the loss of long-term repopulating potential in ex vivo culture for genetic manipulation and the loss to peripheral organs during homing, both of which reduce GM HSC engraftment.

Homing and engraftment of sufficient GM HSC are significant rate-limiting steps for the success of gene therapy. Gene therapy has largely failed unless a significantly higher number of GM HSPC are infused than would be infused for non–gene therapy transplants (1–4). Vector integration analysis has shown that long-term repopulation comes from only a very small fraction of the GM HSC transplanted (3, 4). When BM cells are infused i.v., only 5%–30% of HSC home to the BM, while the remainder are lost to the periphery, particularly the lung, liver, and spleen (5, 6). Homing losses are further compounded in gene therapy transplants by the lack of helper cells, which are removed during HSPC enrichment. These helper cells aid in the homing and engraftment process (7–10).

Due to HSC loss during homing following i.v. HSPC transplant, direct transplant of HSPC to the BM has been proposed and tested as a means to achieve higher retention, increasing homing and engraftment when HSC dose is limited, as is the case with umbilical cord blood (UCB) transplants. This theory has been tested in animal models by intrafemoral injection and in humans by intra–iliac crest injection. However, results have been inconclusive or not promising enough to warrant standardized clinical usage of direct BM transplant. Most experiments have been performed using UCB HSPC or total nucleated cells, and these studies have produced mixed results. Some studies suggest that direct BM transplant of donor HSPC may reduce time to hematopoietic recovery after transplant by reducing the duration of cytopenia (4, 11–13). However, superiority in long-term engraftment is disputed. Furthermore, some experimental models show poor retention of cells in the BM following an intra-BM (i.b.m.) injection, calling the reliability of the method into question (14–16). Hence, a systematic examination of the mode of delivery of GM adult HSC is needed.

We hypothesized that losses of GM HSC homing can be reduced by a mechanistic understanding of homing and engraftment of adult HSPC that occurs with i.v. and i.b.m. transplant. Understanding these mechanisms would significantly lower the burden, scale, and cost of collecting, processing, and genetically manipulating large numbers of HSPC, increase the efficiency of engraftment of GM HSC, and improve success of genetic therapies.

## Results

### Direct BM transplant enhances hematopoietic progenitor cell (HPC) engraftment but not engraftment of the long-term repopulating HSC.

We first validated intrafemoral injection as an accurate means of i.b.m. delivery. UCB CD34^+^ HSPC were transduced with a luciferase-encoding LV vector and transplanted either i.v. or i.b.m. into irradiated NOD.Cg-Prkdc^scid^ Il2rg^tm1Wjl^/SzJ (NSG) mice. Bioluminescent imaging was performed 1, 2, and 12 weeks after transplant, and transduced cell signal was first detected at 2 weeks when sufficient cell proliferation had occurred. I.b.m. injection of HSPC into the femur primarily localized and retained the GM HSPC in the injected femur, while GM HSPC transplanted i.v. were found in both femurs and in the sternal and rib areas (Supplemental Figure 1A; supplemental material available online with this article; https://doi.org/10.1172/jci.insight.151847DS1). By 12 weeks after transplant, however, engraftment of GM cells was widespread with both i.v. and i.b.m. transplant (Supplemental Figure 1A).

**Figure 1:**
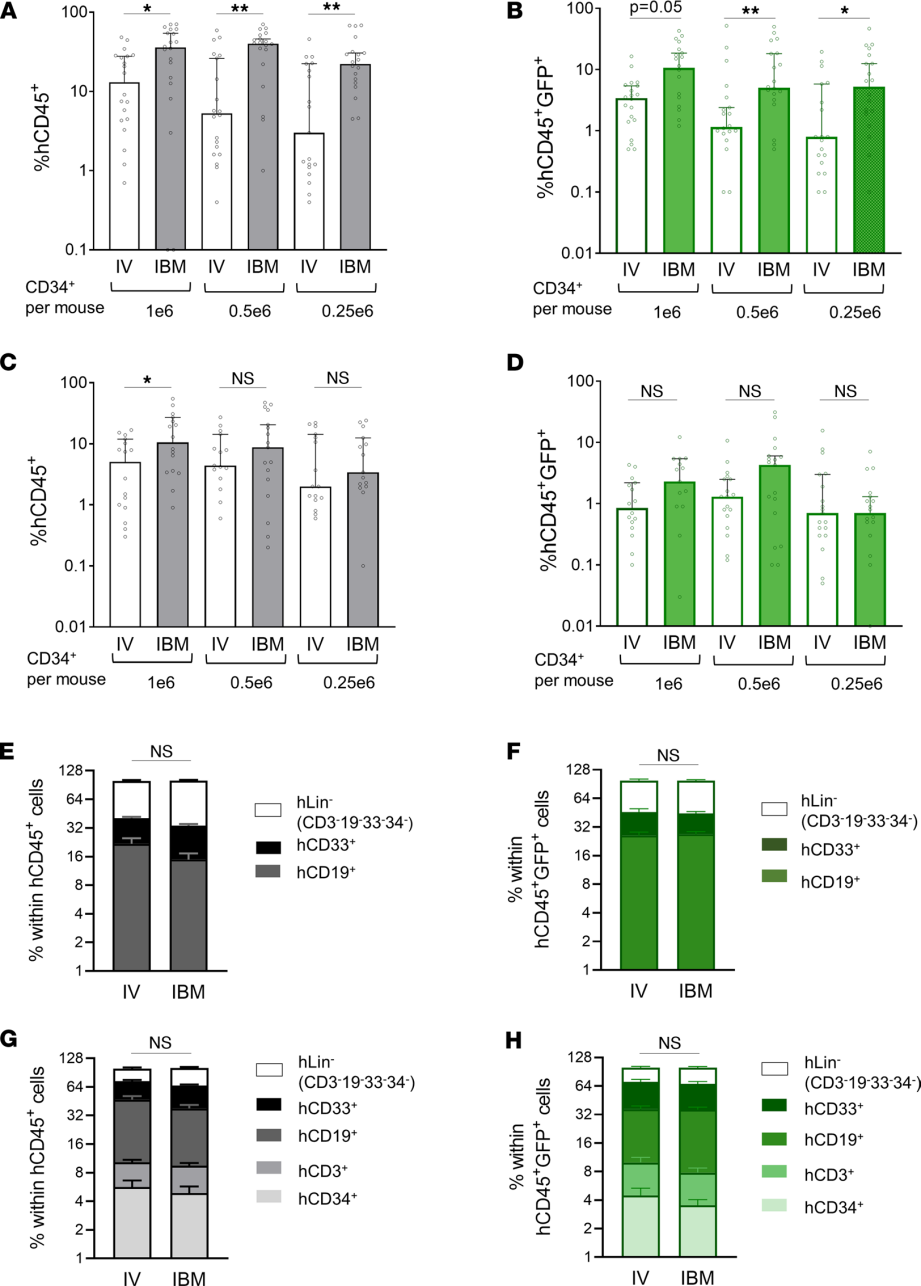
Hematopoietic progenitor cells (HPC) have a preferential engraftment advantage when transplanted in the BM directly in NSG mice. (**A**–**D**) Irradiated NSG mice were transplanted with MPB CD34^+^ HSPC that were transduced with a GFP-encoding LV vector (gene transfer 63%–66%) either i.v. or i.b.m. at the indicated cell doses. Human cell engraftment was analyzed by determining the percentage of human CD45^+^ (hCD45^+^) cells and GM hCD45^+^ (hCD45^+^GFP^+^) cells in the BM obtained via BM aspirate at 12 weeks to assess short-term engraftment (**A** and **B**) and from pooled BM from femurs, tibias, and iliac crests at 24 weeks to determine long-term human engraftment (**C** and **D**). Bars represent median engraftment ± 95% CI. Each symbol represents an individual mouse; *n* = 18–20 mice per cell dose per transplant method. Mice were transplanted using 4 unique MPB donors in 4 experiments; statistical analysis was performed using 1-way ANOVA comparing i.v. and i.b.m. groups at each cell dose with correction for multiple comparisons. (**E**–**H**) Lineage output of the human xenograft. BM was stained with antibodies specific for T cell lineage (hCD3), B cell lineage (hCD19), myeloid lineage (hCD33), and HSPC (hCD34) to determine the lineage output of the transplanted total HSPC (**E** and **G**) and GM HSPC (**F** and **H**) at 12 weeks and 24 weeks after transplant. Statistical analysis was performed using 2-way ANOVA. *P < 0.05, **P < 0.01.

We then determined the short- and long-term engraftment of GM adult human HSPC when transplanted via i.b.m. injection compared with the traditional i.v. transplant. Mobilized peripheral blood (MPB) CD34^+^ HSPC transduced with a GFP-encoding lentivirus vector were transplanted into NSG mice (Supplemental Figure 1B). At 12 weeks after transplant, we performed femoral BM aspirates (from the noninjected femur in the i.b.m. group of mice) and analyzed human cell engraftment (human CD45^+^ cells) in the BM and transduced human cell engraftment (human CD45^+^GFP^+^) (Supplemental Figure 2, A and B). I.b.m. transplantation of HSPC led to significantly higher engraftment of both total and GM human cells in the BM at 12 weeks, particularly when HSPC dose was limited (Figure 1, A and B). However, the long-term (24 weeks after transplant) BM analysis revealed that the engraftment advantage of direct BM transplantation seen at 12 weeks was lost. While the long-term repopulation trended higher with i.b.m. delivery, the improvement over i.v. delivery was not significant (Figure 1, C and D).

BM was also analyzed for human multilineage repopulation at 12 and 24 weeks (representative flow cytometry analyses and gating strategy is shown in Supplemental Figure 2, C–F). At 12 weeks, the human graft was bilineage, primarily composed of B cells and myeloid cells in both total (Figure 1E) and transduced (Figure 1F) human cells, indicating the progeny of a short-term repopulating cell, likely an HPC. At 24 weeks, a multilineage human graft composed of T cells, B cells, myeloid cells, and CD34^+^ HSPC was represented in both the total (Figure 1G) and transduced (Figure 1H) human graft, indicating that, at this time point, the engraftment represented that of a multilineage long-term repopulating cell, likely an HSC.

We then performed a secondary transplant of the human graft from primary mice at 24 weeks (after depleting murine CD45^+^ cells) into secondary NSG mice to definitively compare engraftment of HSC delivered i.v. versus i.b.m. We observed no difference in secondary engraftment of i.b.m. or i.v. transplanted HSC in secondary mice at 12 weeks after transplant — data that were similar to the long-term engraftment results at 6 months in primary mice (Supplemental Figure 3). Hence, the 6-month human primary graft was assumed to be derived from a long-term repopulating HSC that gives rise to a multilineage graft, capable of secondary engraftment.

Of note, at both 12-week and 24-week time points, the GM HSPC engraftment (Figure 1, B and D) followed patterns similar to that of the overall human HSPC engraftment (Figure 1, A and C). Taken together, our data suggest that i.b.m. transplantation of HSPC provided an engraftment advantage to HPC but not to the long-term repopulating HSC.

### I.b.m. transplant improves homing of HSPC via an HSPC-intrinsic mechanism.

**Figure 2:**
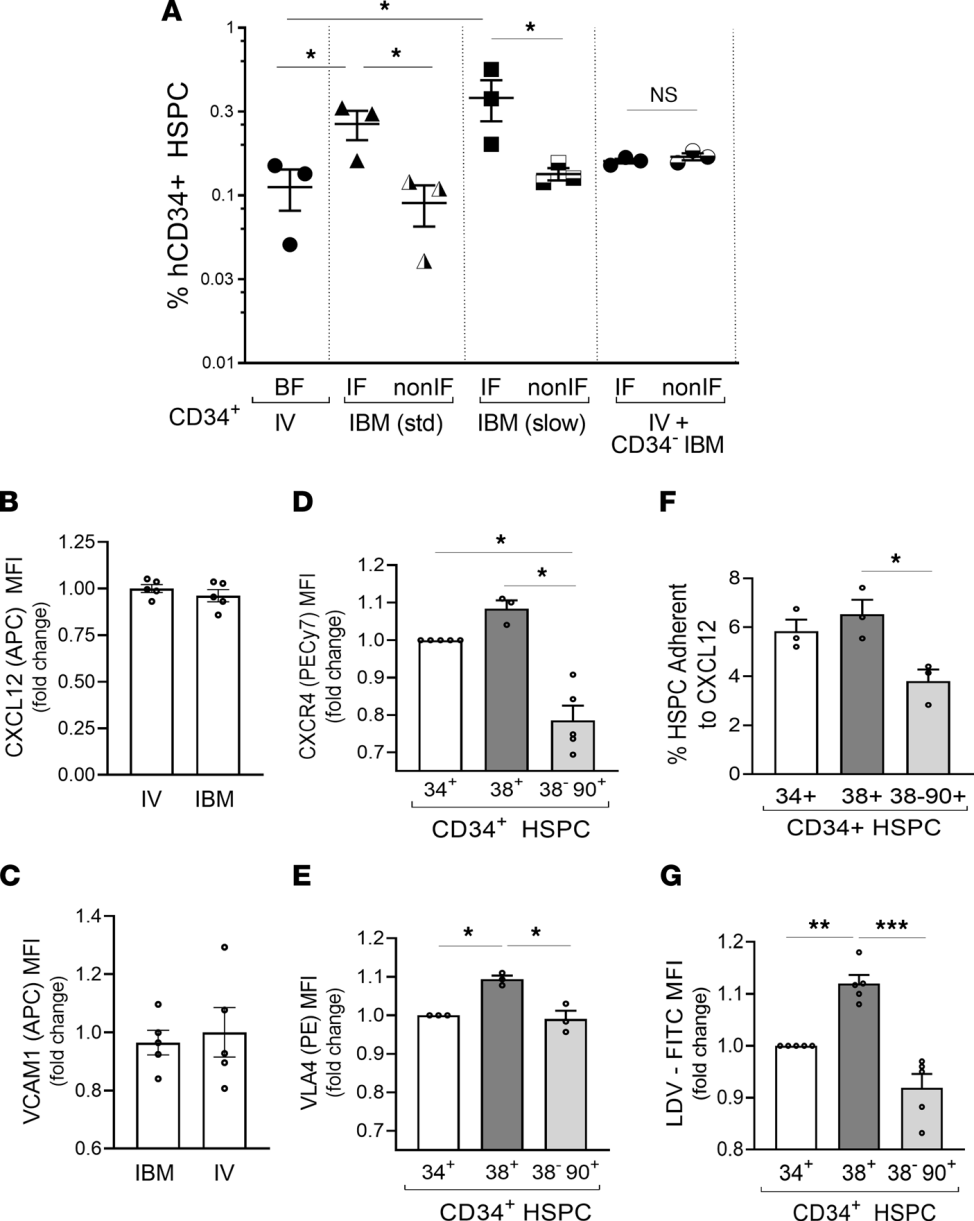
Homing of i.v. and i.b.m. transplanted HSPC and expression of homing receptors on HSC and HPC. (**A**) MPB CD34^+^ HSPC were injected i.v., i.b.m. using a standard syringe, or slow i.b.m. using a Hamilton syringe over 1 minute, and homing of CD34^+^ cells to the BM was analyzed 20–22 hours later. To determine if mechanical pressure in the injected femur alters homing, irradiated CD34^–^ cells were injected i.b.m., and CD34^+^ cells were injected i.v. into the same animal. Homing was determined in the injected femurs (IF) and non-IF with i.b.m. injections, and in both femurs (BF) combined with i.v. injection. Symbols represent individual animals; *n* = 3 mice per group; statistical analysis was performed using 2-way ANOVA. (**B** and **C**) For i.b.m. injections, BM from the IF and non-IF was analyzed for expression of CXCL12 and VCAM1 on mouse stromal cells (hCD45^–^PKH-26^–^, mouse lineage^–^mCD45^–^mCD51^+^ cells). *n* = 5 mice per group; data were normalized to i.v. injected group; statistical analysis was performed by Mann-Whitney *U* test. (**D** and **E**) CXCR4 and VLA4 homing receptor expression of CD34^+^ HSPC, CD34^+^CD38^+^ HPC, and CD34^+^CD38^–^CD90^+^ HSC-enriched populations. Data were normalized to CXCR4 and VLA4 expression on CD34^+^ HSPC. Symbols represent unique MPB donors; *n* = 5 per group; statistical analysis was performed using ANOVA. (**F**) Adhesion of CD34^+^ HSPC, CD34^+^CD38^+^ HPC, and CD34^+^CD38^–^CD90^+^ HSC to CXCL12. Symbols represent unique MPB donors; *n* = 5 per group; statistical analysis was performed using ANOVA. (**G**) Binding of CD34^+^ HSPC, CD34^+^CD38^+^ HPC, and CD34^+^CD38^–^CD90^+^ HSC to the VLA4 affinity detection ligand LDV-FITC. Data were normalized to LDV-FITC expression in CD34^+^ HSPC. Symbols represent unique MPB donors; *n* = 3 per group; statistical analysis was performed using 1-way ANOVA. *P < 0.05, **P < 0.01, ***P < 0.001. In **B**–**E** and **G**, data were normalized to the indicated group, to account for variability in MFI between different experiments/donors.

We next investigated if there was a specific retention or homing advantage to HPC delivered locally into the BM. It was shown that intrafemoral injection of HSPC in rodents results in the majority of cells leaving the BM into circulation within the first 15 seconds but that the injected HSPC home back much more efficiently into the noninjected BM than cells delivered by i.v. injection (15). In a porcine model that better simulates human physiology and scale, Pantin et al. showed that HSPC are retained in the injected BM by lowering the injection volume and injection rate (14). We therefore determined the retention and homing of HSPC in our human engraftment model via 4 modes of transplant of MPB CD34^+^ HSPC: (a) i.v. injection of CD34^+^ HSPC by tail vein, (b) i.b.m. delivery of CD34^+^ HSPC, (c) slow i.b.m. delivery of CD34^+^ HSPC over 1 minute using a Hamilton syringe, and (d) a sham i.b.m. delivery of irradiated CD34^–^ cells followed by a tail vein injection of CD34^+^ HSPC. The latter was done to determine whether the i.b.m. injection induced mechanical/shear stress that altered the BM microenvironment, allowing superior retention and homing of i.v. injected cells in the injected femur. Twenty hours after infusion, mice were sacrificed. For i.b.m. injections, retention of HSPC was determined in the femurs separately; for i.v. injections, homing was determined in both femurs combined (Figure 2A). To determine the accuracy of the injections and explain the homing of i.b.m.-injected HSPC to the contralateral femur, we also labeled CD34^+^ HSPC with PKH-26 (so that rare populations can be clearly identified in blood without any background seen with antibody staining; Supplemental Figure 4, B–D) and analyzed their presence in blood after i.v. and i.b.m. injections. While a small number of HSPC were in circulation after i.b.m. injections at 6 and 22 hours after transplant, a much higher number of CD34^+^ HSPC were in circulation at 6 hours and 22 hours after i.v. transplant. The injected femur (IF) of i.b.m. mice had significantly higher human CD34^+^ HSPC content than did the non-IF. The non-IF had similar homing of HSPC into BM as that seen in the i.v. injected mice. Slow i.b.m. delivery of HSPC did not alter the HSPC patterns as compared with the standard i.b.m. injections, indicating that retention and homing of CD34^+^ HSPC in the IF was not impacted by delivery pressure if a small volume is injected in the mice. Additionally, the sham injection of CD34^–^ cells did not alter homing behavior of i.v. injected CD34^+^ HSPC in the sham IF, which had similar homing as that seen in the nonsham IF, suggesting that an HSPC-intrinsic rather than microenvironment mechanism likely mediates this effect (Figure 2A). Consistent with this observation, the mechanical pressure of i.b.m. delivery did not alter the expression of stromal cell ligands for the 2 most well characterized homing receptors on HSPC.

BM stromal cells (mouse lineage^–^, human CD45^–^PKH^–^, mouse CD51^+^ cells; human HSPC were PKH labeled; gating for murine stromal cells is shown in Supplemental Figure 4A) from the IF and non-IF were studied to determine if expression of CXCL12, the ligand for CXCR4, and VCAM1, the ligand for VLA4, changed in response to pressure increases associated with injection through the BM. Expression of CXCL12 (Figure 2B) and VCAM1 (Figure 2C) examined in the murine and human nonhematopoietic mouse stromal cell population was not significantly different in the BM stroma in the IF compared with the non-IF, further suggesting that microenvironment changes due to injection were not a significant factor in observed differential engraftment behavior and that the difference was likely cell intrinsic.

### Differential homing receptor expression on HPC and HSC.

We postulated that cell-intrinsic differences of homing receptor expression between HPC and HSC may explain the differential short- and long-term engraftment. We determined the expression of the 2 major HSPC homing and retention receptors, CXCR4 and VLA4, on CD34^+^CD38^+^ cells, which are largely HPC, and CD34^+^CD38^–^CD90^+^ cells, a population highly enriched in HSC. We found that CD34^+^CD38^+^ HPC had significantly higher surface expression of VLA4 and CXCR4 than did CD34^+^CD38^–^CD90^+^ HSC (Figure 2, D and E). Accordingly, CD34^+^CD38^+^ HPC functionally adhered more avidly to a CXCL12-coated plate than did the CD34^+^CD38^–^CD90^+^ HSC-enriched cells (Figure 2F). Furthermore, CD34^+^CD38^+^ HPC had increased VLA4-ligand binding affinity compared with CD34^+^CD38^–^CD90^+^ HSC (Figure 2G). Overall, these results suggest that increased VLA4 and CXCR4 expression on HPC may be involved in conferring a retention and homing advantage to HPC over HSC following i.b.m. administration, and therefore, the engraftment advantage was short lived.

Expression of both VLA4 and CXCR4 on CD34^+^ HSPC is upregulated by stem cell factor and Flt-3 ligand (17–24), cytokines that are used in ex vivo cultures for genetic manipulation; indeed, both VLA4 and CXCR4 expression on CD34^+^ HSPC increased with increasing time in culture (Supplemental Figure 5, A–D). Expression of VLA4 and CXCR4 was highest in CD34^+^ HSPC in G_2_M phases and S phase in contrast to that on the quiescent HSPC, and as expected, more CD34^+^CD38^+^ HPC were cycling, while a great majority of CD34^+^CD38^–^CD90^+^ HSC were in G0/G1 phase (Supplemental Figure 5, E–H). To determine if CXCR4 expression was indeed cell cycle dependent, we examined CXCR4 and VLA4 expression levels on HSC and HPC that were in G0 phase of cell cycle. Indeed, HPC and HSC in G0 phase had similar CXCR4 and VLA4 expression (Supplemental Figure 5, I and J).

However, while LV transduction significantly increased VLA4 expression on the GM HSPC (GFP^+^) cells, transduction of HSPC did not have an effect on surface expression of CXCR4 (Supplemental Figure 5, K and L), much like the engraftment data of transduced and untransduced HSPC (Figure 1, A–D).

### Differential CXCR4 expression provides a homing advantage to HPC.

**Figure 3:**
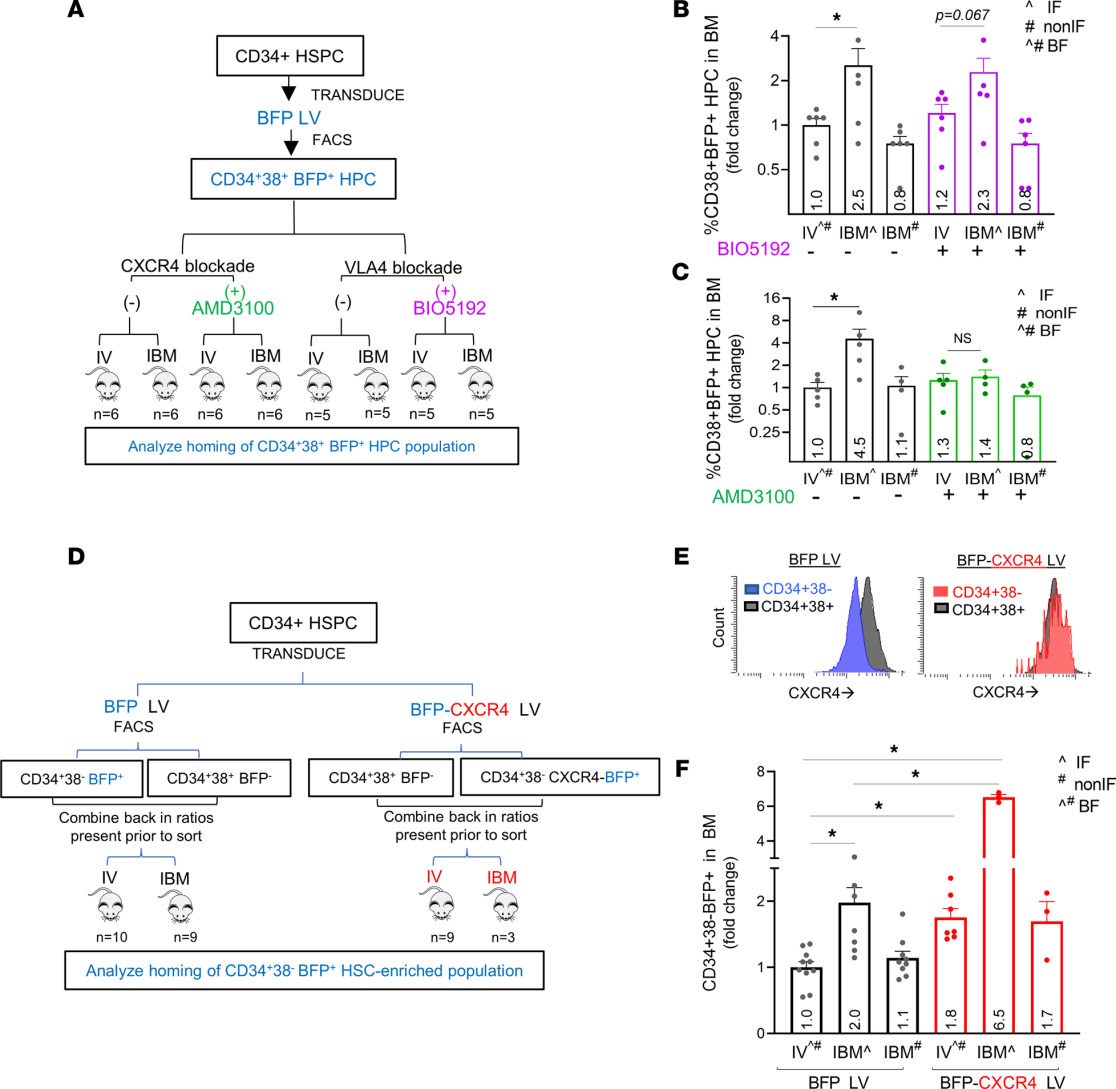
The level of expression of CXCR4, not VLA4, on HSPC directs homing into BM with i.b.m. transplants. (**A**–**C**) High CXCR4 expression on HPC mediates their preferential homing locally with i.b.m. transplant; CD34^+^ HSPC were transduced with a BFP LV vector, and the CD34^+^CD38^+^BFP^+^ HPC were sorted by flow cytometry 72 hours after gene transfer. HPC were blocked with AMD3100 (CXCR4 antagonist) or BIO5192 (VLA4 antagonist) before i.v. or i.b.m. delivery (**A**). Homing of hCD34^+^ cells was analyzed in the injected femur (IF) and non-IF for i.b.m. injected mice and in both femurs combined in i.v. injected mice. Data were normalized to i.v. injected control CD34^+^CD38^+^ cells, and the fold increase is indicated within the bar (**B** and **C**). *n* = 5–6 mice per group; statistical analysis was performed by 1-way ANOVA. (**D**–**F**) Induction of high CXCR4 expression on CD34^+^CD38^–^ HSC enriched population via gene transfer confers them with a homing advantage despite higher abundance of CD34^+^CD38^+^ HPC. CD34^+^ HSPC were transduced with a BFP LV vector or a BFP-CXCR4 LV vector and sorted for transduced CD34^+^CD38^–^BFP^+^ cells and untransduced CD34^+^CD38^+^BFP^–^ HPC at 72 hours. Transduced CD34^+^CD38^–^ cells were mixed with untransduced CD34^+^CD38^+^ cells and transplanted i.v. or i.b.m., and homing of CD34^+^CD38^–^BFP^+^ cells into BM was analyzed at 20–22 hours (**D**). CXCR4 expression on injected cells at time of transplant is shown and the MFI on injected cells at time of transplant was as follows: CD34^+^CD38^–^ control, 16,930; CD34^+^CD38^+^ control, 35,098; and CD34^+^CD38^–^CXCR4 transduced, 37,453. (**E**). Homing in BM is shown (**F**). *n* = 3–10 mice per experimental arm; statistical analysis was performed using 1-way ANOVA. *P < 0.05.

Since HPC have much higher expression of both CXCR4 and VLA4 compared with HSC, we sought to determine whether both or one of them promoted HPC retention and homing with direct i.b.m. transplantation. We transduced CD34^+^ cells with a blue fluorescent protein (BFP) encoding LV vector and sorted the CD34^+^CD38^+^BFP^+^ HPC by flow cytometry. VLA4 and CXCR4 were blocked by exposing the sorted cells to BIO5192 (a VLA4 inhibitor) and AMD3100 (a CXCR4 antagonist), respectively, before injection into irradiated NSG mice (Figure 3A) to determine their role in retention/homing of CD34^+^CD38^+^ HPC into the BM by i.b.m. versus i.v. administration. In the homing model, control CD34^+^CD38^+^ HPC, which were not subjected to CXCR4 or VLA4 blockade, homed to the BM at significantly higher numbers in the IF with i.b.m. delivery than in the non-IF or in BF with i.v. delivery (Figure 3, B and C), similar to the results seen with homing of CD34^+^ HSPC (Figure 2A). Blockade of VLA4 did not alter the CD34^+^CD38^+^ HPC homing pattern, as compared with the corresponding controls (Figure 3B), indicating that the VLA4 homing receptor was not the major contributor to the higher retention/homing of HPC into the injected BM. However, blockade of CXCR4 with AMD3100 significantly reduced human HPC content in the IF to homing levels seen with i.v. delivery or homing levels in the non-IF (Figure 3C). Notably, homing of i.v. injected cells was not blocked with AMD3100, suggesting that other homing receptors may play a role here. Taken together, these data show that higher expression of CXCR4, not VLA4, on HPC confers a competitive homing advantage.

An alternative explanation for the competitive homing and engraftment advantage of HPC could be that they comprise the vast majority of CD34^+^ HSPC (98%–99%), while HSC are a small minority. We experimentally conferred the HSC-enriched CD34^+^CD38^–^ population with slightly higher CXCR4 expression than the CD34^+^CD38^+^ HPC (Figure 3D). CD34^+^ HSPC were transduced with a BFP-CXCR4–encoding LV vector or a BFP-encoding LV control vector, and CD34^+^CD38^–^BFP^+^ HSC-enriched cells were sorted along with untransduced CD34^+^CD38^+^BFP^–^ HPC. We assessed the CXCR4 expression on these populations before transplant. In control cells, CXCR4 expression on the more primitive CD34^+^CD38^–^ cells was much lower (mean fluorescence intensity [MFI] 16,930) than on CD34^+^CD38^+^ HPC (MFI 35,098) (Figure 3E), consistent with prior data (Figure 2D). In contrast, CXCR4 expression on the HSC-enriched CD34^+^CD38^–^BFP^+^ cells transduced with the BFP-CXCR4 LV vector was now higher (MFI 37,453) than the CD34^+^CD38^+^ HPC (Figure 3E). We then mixed the sorted BFP^–^ HPC population with the BFP^+^CD34^+^CD38^–^ CXCR4^hi^ HSC-enriched cell population in ratios seen in CD34^+^ HSPC, prior to sorting, and transplanted them either i.v. or i.b.m.; we then assessed homing of the CD34^+^CD38^–^BFP^+^CXCR4^hi^ HSC-enriched cells. Control CD34^+^CD38^–^BFP^+^ cells were also mixed with CD34^+^CD38^+^BFP^–^ HPC and transplanted i.v. or i.b.m. (Figure 3D), and their homing into BM was assessed. We observed that, even when CD34^+^CD38^–^BFP^+^CXCR4^hi^ cells were in the minority, they were able to outcompete the CD34^+^CD38^–^BFP^–^ HPC that were in the majority, when injected i.b.m. Furthermore, even i.v. administration of CD34^+^CD38^–^BFP^+^CXCR4^hi^ cells mixed with a majority of CD34^+^CD38^+^ HPC led to higher homing levels of CD34^+^CD38^–^ cells at levels comparable with i.b.m. delivery. Hence, when the CD34^+^CD38^–^ HSC-enriched population was conferred with higher CXCR4 expression than HPC, it had a higher homing advantage to the BM following i.v. transplantation and a remarkably improved homing advantage following direct i.b.m. transplantation (Figure 3F). Taken together, these data suggest that: (a) CXCR4, not VLA4, plays a major role in the differential retention and homing (and therefore likely engraftment) of HPC when delivered i.b.m.; (b) cells highly enriched in HSC, when experimentally endowed with higher CXCR4 expression, have a homing advantage over HPC; and (c) the sheer abundance of HPC did not give them a competitive homing advantage.

### i.b.m. delivery of an HSC-enriched population enhances long-term repopulation compared with i.v. delivery.

**Figure 4:**
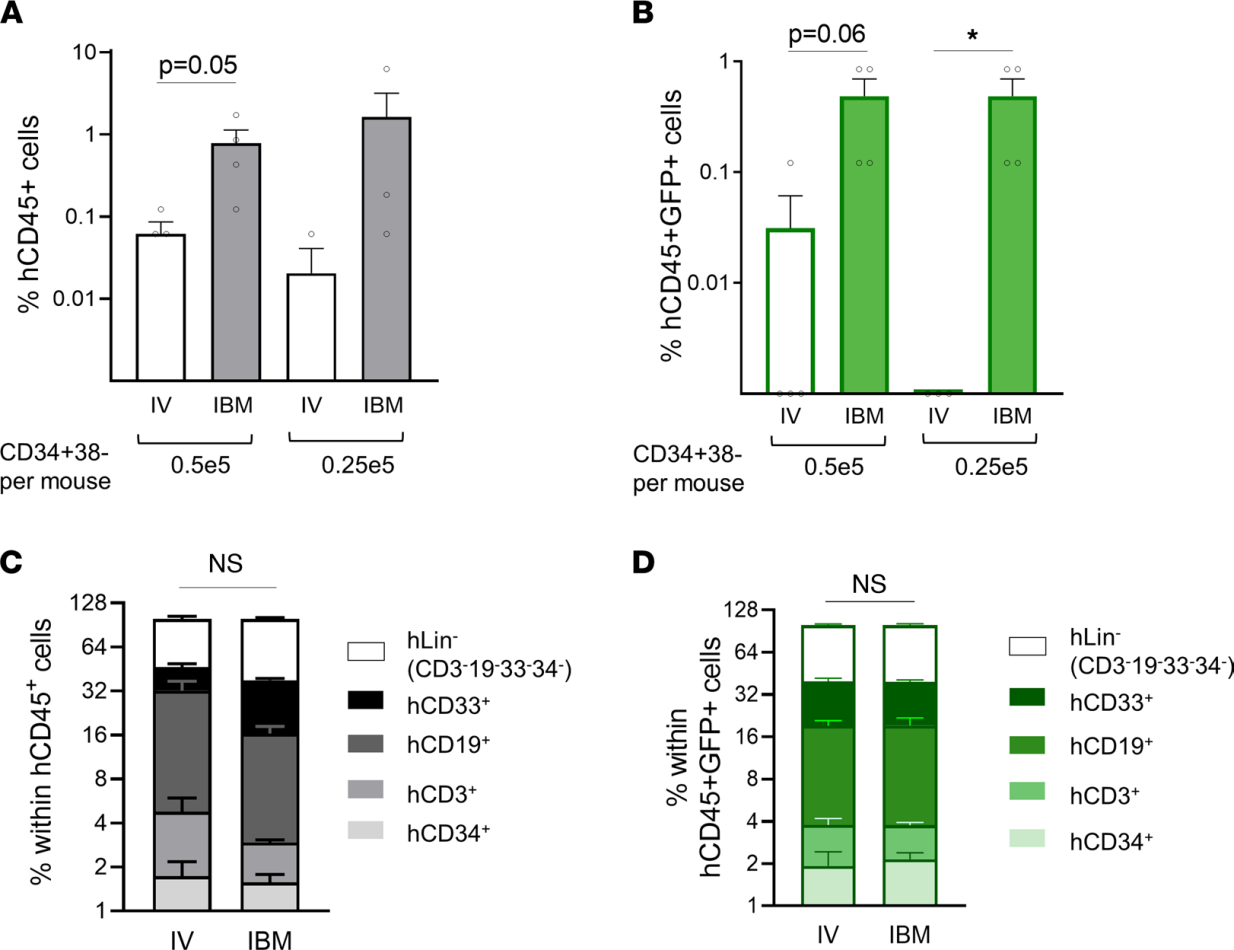
I.b.m. transplant of an HSC-enriched population removes the competition from HPC and enhances long-term repopulation compared with i.v. delivery. CD34^+^CD38^–^ HSC-enriched cells were obtained using immunomagnetic sorting, transduced with a GFP LV vector (gene transfer efficiency was 69%), and transplanted into NSG mice i.v. or i.b.m. in 2 limiting dilution doses. (**A** and **B**) Long-term (24 week) engraftment of total (hCD45^+^) and GM human (hCD45^+^GFP^+^) cells is shown. Symbols represent individual animals; *n* = 3–4 mice per treatment condition; statistical analysis was performed by comparing the modes of delivery comparing the different cell doses using 1-way ANOVA. *P < 0.05. (**C** and **D**) Lineage output at 24 weeks shows that the long-term human graft was multilineage, composed of B, T, and myeloid cells and CD34^+^ HSPC. Statistical analysis was performed using 2-way ANOVA.

Based on these results, we hypothesized that removal of the high CXCR4-expressing CD34^+^CD38^+^ HPC followed by transduction and transplantation of CD34^+^CD38^–^ HSC-enriched cell population would confer i.b.m.-delivered HSC a competitive advantage to long-term engraftment in the BM niche. We magnetically depleted CD38^+^ HPC from the CD34^+^ HSPC population and transplanted the CD34^+^CD38^–^ HSC-enriched population in NSG mice i.v. and i.b.m. We achieved 92% purity in CD34^+^CD38^–^ cells using the CD34^+^CD38^–^ Isolation Kit. Mice were analyzed for long-term engraftment at 24 weeks after transplant. i.b.m. administration led to significantly increased long-term multilineage engraftment compared with i.v. administration (Figure 4, A and B), suggesting competition between HPC and HSC with i.b.m. delivery that is removed with HPC depletion. In fact, this advantage was significant in the GM cells where cell doses were limited (Figure 4B). Lineage analysis of the human graft showed that the human cells (total and GM) were multilineage (Figure 4, C and D).

### Transient high CXCR4 expression confers a homing and engraftment advantage to CD34^+^CD38^–^ long-term repopulating cells.

We next sought to increase CXCR4 transiently on GM long-term repopulating cells (LTRC) to give them a competitive homing and engraftment advantage over HPC. Several treatments can be added to culture to achieve higher CXCR4 expression, specifically CD26 inhibition, mild hyperthermia, prostaglandin E2 (PGE2), glucocorticoid treatment, and HDAC inhibition (25–31). However, they increase CXCR4 expression on all HSPC (GM and non-GM HSPC) in culture. Increasing CXCR4 expression only on GM HSC is desirable but poses challenges. CXCR4 cannot be carried on an integrating vector (as was done in the results shown in Figure 3, D–F, and by Brenner et al; ref. 32) because the long-term clinical consequences of this approach are questionable. Specifically, permanent upregulation of CXCR4 leads to myelokathexis and a primary immunodeficiency caused by a hyper functional CXCR4 receptor (33). Regulated expression by placing CXCR4 as an inducible cassette within the transgene vector is not feasible due to low vector titer resulting from large insert size.

We wanted to confer transient high CXCR4 expression to only GM CD34^+^CD38^–^ HSC-enriched cells to develop an approach that would be clinically translatable. This HSC-enriched population was chosen instead of HSC because magnetic sorting for this cell population is now commercially available, efforts to make it clinically scalable are underway, and CD34^+^CD38^–^ cells have been shown to contain HSC with long-term repopulating ability (34). Our strategy was to deliver CXCR4 as a protein transiently within the LV vector particle so that GM HSPC obtain the homing advantage and, therefore, have higher engraftment than non-GM HSPC.

**Figure 5:**
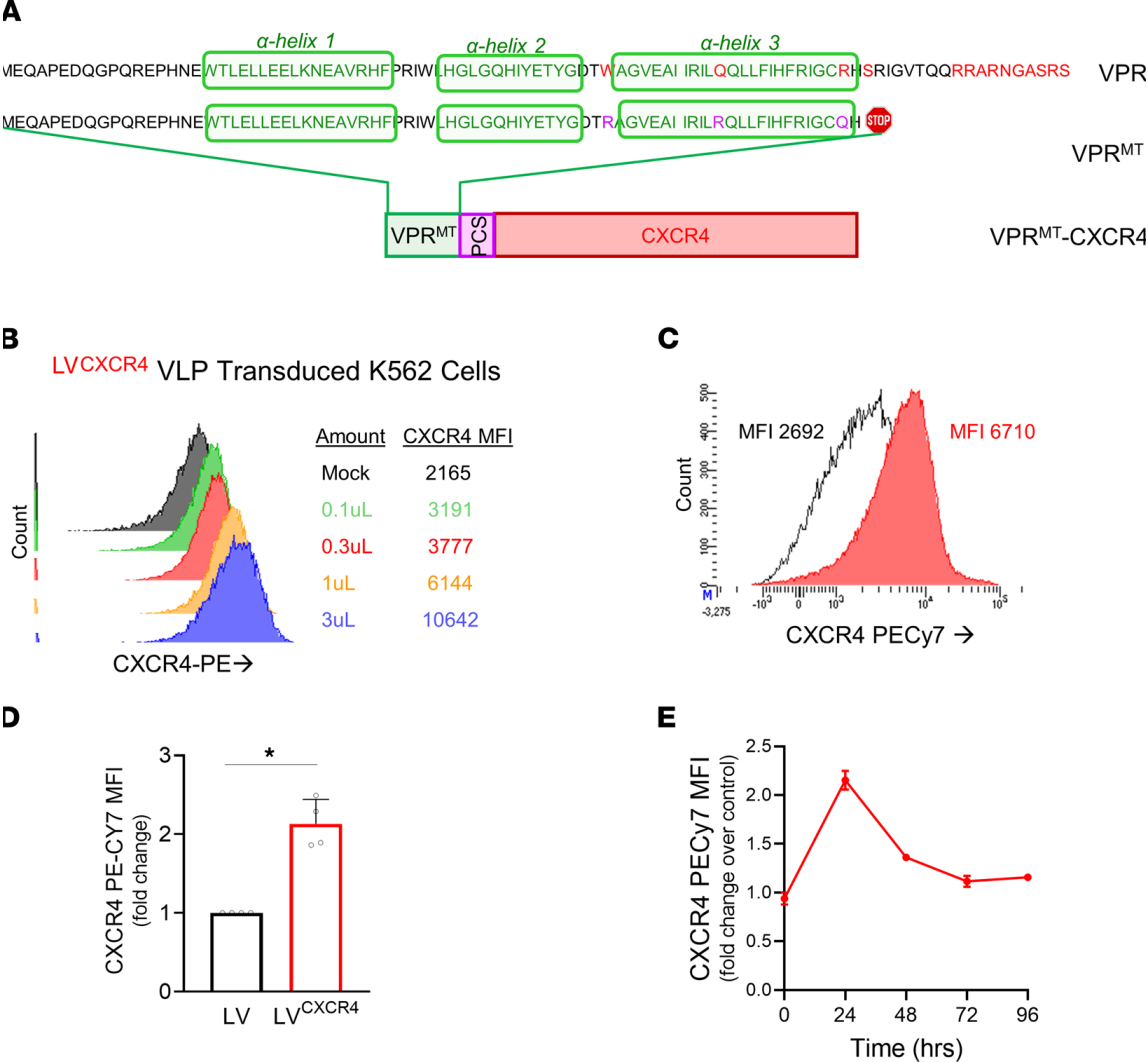
Development of CXCR4 protein delivery within the lentiviral (LV) vector. (**A**) Construct design. The aa sequence of VPR and its mutant version generated is shown. VPR, a small HIV-1 accessory protein, is carried in viral particles bound to gag via residues in its central region that fold into 3 α-helices. The C-terminal domain has 6 arginine residues that potentiate nuclear localization, G_2_M arrest, and apoptosis. S79 phosphorylation is important for cell cycle arrest. We truncated VPR at the 78 aa and mutated VPR^R77Q^. W54 and Q65 interacts with DNA damage response (DDR) proteins via UNG2 and DCAF and were mutated to VPR^W54R^ and VPR^Q65R^. The triple mutated and truncated VPR (VPR^MT^) would lack pathogenicity (residues known to be associated with VPR toxicity are highlighted in red) but allow binding to gag. VPR^MT^ was fused to CXCR4 cDNA via the HIV-1 protease cleavage site (PCS) to generate VPR^MT^-CXCR4. (**B**) K562 cells were transduced with LV^CXCR4^ vector-like particles (VLP; empty vector particles lacking the vector genome) at increasing particle concentration and analyzed for CXCR4 expression using flow cytometry. Mean fluorescence intensity (MFI) of CXCR4 is listed against volume of VLP added. (**C** and **D**) A GFP-encoding LV was either packaged using standard packaging plasmids (LV, black) or packaged with VPR^MT^-CXCR4 plasmid in addition in order to package the CXCR protein attached to the LV capsid (LV^CXCR4^, red). CXCR4 expression on MPB CD34^+^ cells transduced with LV^CXCR4^ vector compared with cells transduced with the control LV vector 24 hours following gene transfer is shown. *n* = 4; statistical analysis was performed by Mann-Whitney *U* test. (**E**) The time course of CXCR4 expression in LV^CXCR4^ HSPC, normalized to that of control LV HSPC, is shown, with expression peaking at 24 hours that returns to baseline by 72 hours. Symbols represent individual MPB donors; *n* = 3.

Viral protein R (VPR) is a well-characterized small HIV-1 accessory protein that is unique in being present in HIV-1 virions bound to the capsid protein, gag, via residues in its central region that folds into 3 α-helices (Figure 5A). These are flanked by unstructured N- and C-terminal domains. VPR mediates early T cell toxicity upon viral cell entry and is therefore excluded from the LV vector packaging plasmids. It mediates its toxicity via nuclear localization domains, and its specific residues that cause cell cycle arrest and apoptosis, as well as interact in the DNA damage response (DDR), have been well characterized and highlighted in Figure 5A (VPR) (35–41). The C-terminal domain imparts protein stability and has 6 arginine residues between positions 73 and 96 that potentiate nuclear localization, G_2_M arrest, and apoptosis. S79 phosphorylation is important for cell cycle arrest. We therefore truncated VPR at the 78 aa and VPR^R77Q^ mutation was made in the third helix. The third helix forms a leucine zipper–like motif that interacts with DDR proteins and UNG2 via W54, and the ubiquitin-proteasome complex via DCAF via Q65, and induces DDR signaling. VPR^W54R^ and VPR^Q65R^ mutations were also made to abrogate binding to UNG2 and DCAF, preventing DDR, and to eliminate all changes in the proteome normally triggered by VPR upon viral entry, respectively. We designed this mutated (W54R, Q65R, and R77Q) and truncated (78 aa) version of VPR (VPR^MT^) to retain its folding, oligomerization and gag/capsid binding domains but to remove residues and the carboxyl terminal region that mediate cytotoxicity.

Next, VPR^MT^ was fused to CXCR4 cDNA via the HIV-1 protease cleavage site (PCS) to generate VPR^MT^-CXCR4. The PCS is recognized by the HIV protease, an enzyme present in LV vectors along with reverse transcriptase and integrase enzymes. All three of these enzyme proteins are encoded by the Pol gene present in the packaging plasmids (Supplemental Figure 6A). We fused VPR^MT^ to CXCR4 cDNA using a PCS of lentiviruses so that CXCR4 is released from VPR^MT^ upon cell entry and can, therefore, express on the cell surface.

Currently, standard LV vectors are generated by transfecting 293T cells with (a) the packaging plasmids that provide the proteins that form the viral capsid (encoded by Gag), enzymes (reverse transcriptase, integrase, and protease; encoded by Pol), and envelope proteins to form the viral particle and (b) the vector genome plasmid that encodes the transgene mRNA. Only the vector genome plasmid has the encapsidation signal allowing packaging of its genetic material/RNA into the vector particles. Vector particles are assembled from the proteins encoded by the packaging plasmids, and no genetic material from the packaging plasmids is encapsidated into viral particles (Supplemental Figure 6A).

Adding the VPR^MT^-CXCR4 as an additional packaging plasmid should result in packaging this fusion protein within the LV vector particle bound to the capsid protein (Supplemental Figure 6C), so that when the vector transduces cells, the fusion protein would be released in transduced cells and cleaved by the protease at the PCS, releasing CXCR4 to come to the cell surface. This strategy would allow delivery of CXCR4 as a protein only on cells transduced by the LV vector, while the vector delivers and integrates the therapeutic transgene into the cellular genome.

We first produced vector-like particles (VLP) or empty LV^CXCR4^ particles, that are generated without cotransfecting the vector transgene plasmid. Here, we used the VPR^MT^-CXCR4 plasmid in addition to the standard packaging plasmids (gag-Pol, Rev, and VSV-G) (Supplemental Figure 6B) to determine if this strategy results in cell surface expression of CXCR4 in the cells transduced with LV^CXCR4^ VLP that contain no genetic material. We transduced K562 cells (ATCC), which normally do not express CXCR4, at increasing concentrations of LV^CXCR4^ VLP. CXCR4 surface expression on the transduced K562 cells increased in a VLP dose-dependent manner, demonstrating that VPR^MT^-CXCR4 protein, delivered attached to the lentivirus vector capsid protein from the LV^CXCR4^ VLP, indeed resulted in cell surface expression of CXCR4 (Figure 5B).

Next, we generated a GFP vector either packaged with VPR^MT^-CXCR4 and other standard LV packaging plasmids (GFP LV^CXCR4^ vector) or packaged using only the standard LV packaging plasmids (GFP LV vector) (Supplemental Figure 6C), and we tested both vectors in MPB CD34^+^ HSPC. In primary MPB CD34^+^ HSPC, transduction with the GFP LV^CXCR4^ vector was sufficient to induce significantly higher CXCR4 expression (a 2-fold higher CXCR4 MFI) on CD34^+^ HSPC as compared with CD34^+^ HSPC transduced with the control GFP LV vector (Figure 5, C and D). Furthermore, since the CXCR4 was delivered as a protein, the expression of CXCR4 in the GFP LV^CXCR4^–transduced CD34^+^ HSPC was transient, peaking at 24 hours and returning to baseline levels by 72 hours after transduction (Figure 5E).

Notably, in the VPR^MT^-CXCR4 fusion construct, VPR domains/residues known to cause cytotoxicity to T cells were removed, while portions were retained that allow proper protein folding and capsid-binding. We wanted to ensure that this fusion construct delivered via LV^CXCR4^ was not associated with increased cytotoxicity to HSPC compared with a standard control LV vector. We therefore transduced MPB CD34^+^ cells with GFP LV^CXCR4^ and GFP LV, and we assessed for the known toxicities of VPR: G_2_M cell cycle arrest, cell viability, apoptosis, and induction of DDR in CD34^+^ HSPC and CD34^+^CD38^–^CD90^+^ HSC (Supplemental Figure 7). The VPR^MT^-CXCR4 delivery via the LV^CXCR4^ showed no toxicity as compared with mock-transduced and standard LV-transduced HSPC and HSC. Hence, this truncated and mutated VPR^MT^-CXCR4 delivered CXCR4 to the surface of LV transduced cells, expressed transiently, without inducing cytotoxicity.

**Figure 6:**
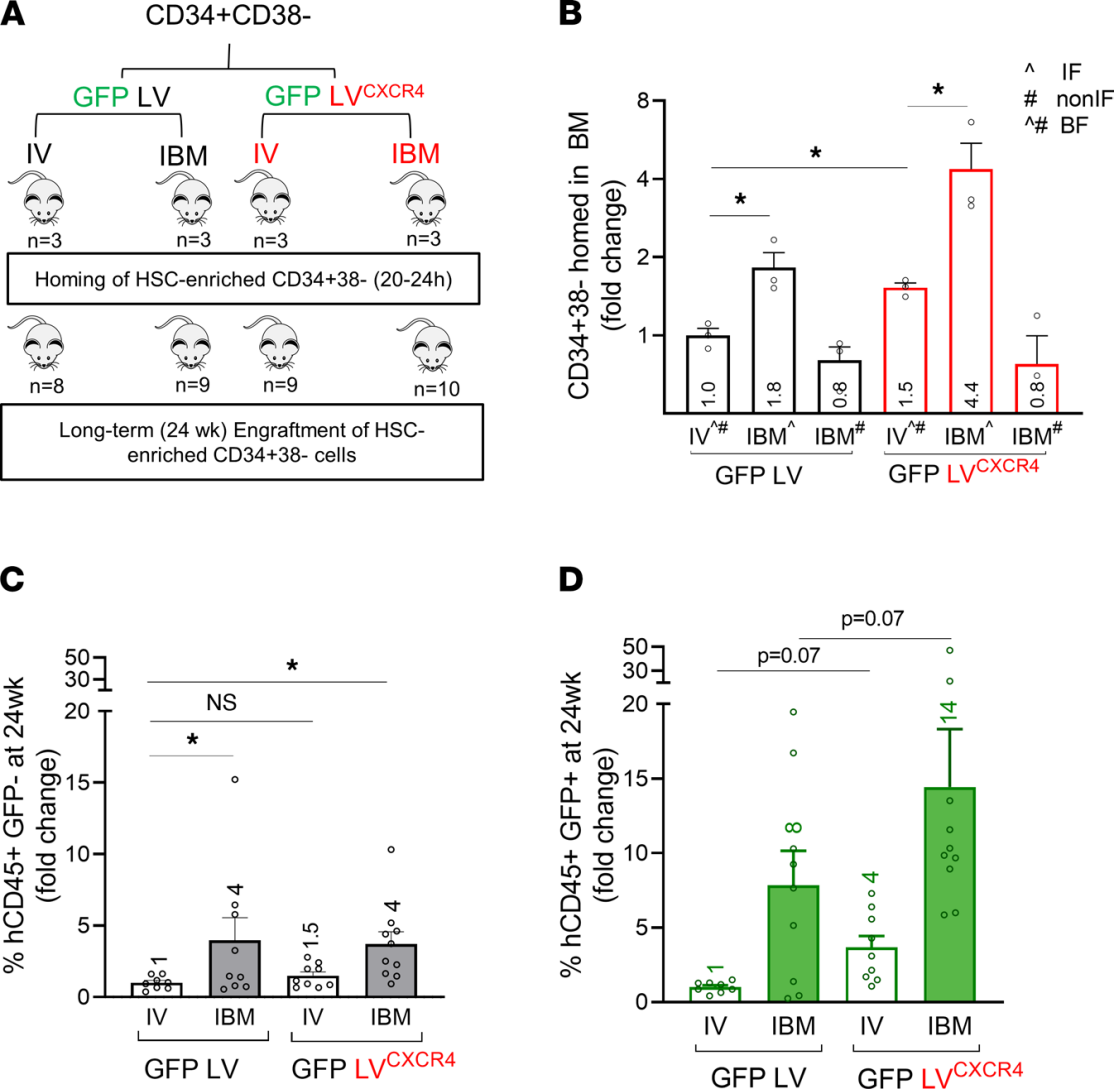
Transiently increased expression of CXCR4 on GM CD34^+^CD38^–^ cells via protein delivery within the LV vector particle significantly increases their homing and long-term engraftment. Experimental schema for assessment of homing and engraftment of CD34^+^CD38^–^ cells transduced with GFP LV or GFP LV^CXCR4^ that were transplanted into NSG mice either via i.v. or i.b.m. delivery. (**A**) The number of animals used for the homing experiment and engraftment experiments is indicated under each experimental arm. (**B**) Homing of CD34^+^CD38^–^ cells in the BM. Data were normalized to i.v. injected control CD34^+^CD38^–^ cells, and the fold increase is indicated within the bar. Symbols represent individual mice; statistical analysis was performed using ANOVA. (**C** and **D**) Long-term engraftment of CD34^+^CD38^–^ cells was assessed by determining the percentage of human CD45^+^GFP^–^ cells (**C**) and human CD45^+^GFP^+^ (**D**) cells 24 weeks following transplant. Data were normalized to i.v. transplanted GFP LV transduced CD34^+^CD38^–^ cells, and the fold increase is indicated within the bar. Gene transfer was 60% with GFP LV and 52% with GFP LV^CXCR4^. Symbols represent individual mice; statistical analysis was performed by 1-way ANOVA. *P < 0.05, **P < 0.01.

We then transduced CD34^+^CD38^–^ HSC-enriched cells with the GFP LV^CXCR4^ vector or a control GFP LV vector and transplanted them i.v. or i.b.m. in NSG mice to assess homing and engraftment (Figure 6A). For assessment of homing, CD34^+^CD38^–^ HSC-enriched cells were labeled with PKH-26 before i.v. or i.b.m. delivery into irradiated NSG mice to allow accurate detection because GFP expression had not yet peaked (it takes 36–60 hours for peak transgene expression following LV after entry, reverse transcription, and integration into the cellular genome, followed by transcription and protein expression of GFP). Homing to the BM was assessed at 20 hours after delivery (Figure 6B). There was significantly increased CD34^+^CD38^–^ HSC-enriched cell homing to the BM after i.v. delivery of GFP LV^CXCR4^ vector–transduced CD34^+^CD38^–^ cells as compared with the i.v. delivery of control GFP LV vector–transduced CD34^+^CD38^–^ cells. In fact, homing of GFP LV^CXCR4^–transduced CD34^+^CD38^–^ cells delivered i.v. was at levels of homing comparable with those in the IF of the i.b.m. GFP LV control mouse group (Figure 6B). Furthermore, within the i.b.m.-injected mice, homing of GFP LV^CXCR4^–transduced CD34^+^CD38^–^ cells to the BM was at more than twice the levels seen in the GFP LV–transduced CD34^+^CD38^–^ HSC-enriched controls in the IF and at more than 4 times that of i.v. delivery (Figure 6B). These data indicate that transient upregulation of CXCR4 confers a strong homing advantage to CD34^+^CD38^–^ HSC-enriched cells when transplanted i.v, and that this homing advantage is even stronger when transplanted directly into BM. The fact that this method of CXCR4 protein delivery utilizes vector packaging and not the vector genome means that any vector genome/transgene can be packaged using this additional fusion plasmid to provide a 2- to 4-fold homing advantage, specifically to GM cells, which in turn may confer higher engraftment to GM HSC.

Next, we transduced CD34^+^CD38^–^ HSC-enriched cells with GFP LV^CXCR4^ vector or GFP LV control vector, and we transplanted them i.v. or i.b.m. in 2 limiting dilutions to irradiated NSG mice to assess engraftment of the long-term repopulating HSC at 6 months after transplant (Figure 6, C and D). The engraftment of GFP LV^CXCR4^ GM CD34^+^CD38^–^ cells transplanted i.v. was significantly (4-fold) higher than GFP LV GM CD34^+^CD38^–^ cells transplanted i.v. (Figure 6C and Supplemental Figure 8, A–D). Following i.b.m. delivery, GFP LV^CXCR4^–transduced CD34^+^CD38^–^ HSC-enriched cells had a nearly 2-fold higher engraftment than i.b.m. delivery of GFP LV–transduced CD34^+^CD38^–^ cells. There was no significant difference in long-term repopulation of untransduced cells between GFP LV and GFP LV^CXCR4^ groups, confirming that the engraftment advantage seen in transduced cells is due to the transient increase in CXCR4 expression (Figure 6C and Supplemental Figure 8, A–D). The slightly higher engraftment of untransduced i.v. transplanted cells (Figure 6C and Supplemental Figure 8C) is likely because LV preparations often contain some defective viral particles, which, like VLP, would deliver CXCR4 protein but not result in an effective transduction (transgene transfer or GFP^+^ cells). Hence, some untransduced cells may have received defective particles, which would still carry the CXCR4 protein. Cells transduced with both GFP LV and GFP LV^CXCR4^ produced full multilineage repopulation at 24 weeks (Supplemental Figure 8, E and F), confirming engraftment of HSC. Taken together, LV vectors used to transduce HSC-enriched cells can be used to confer a homing and engraftment advantage specifically to the transduced HSC-enriched populations. With limiting GM HSC dose, i.b.m. delivery using this method can result in significantly higher long-term engraftment.

## Discussion

Improving the engraftment of GM adult HSC to achieve high levels of engraftment with a limited cell dose is essential to broadening the use of gene therapy. Herein, using a human NSG xenograft model, we show that direct BM transplantation of GM HSPC enhances HPC engraftment but does not significantly improve engraftment of LTRC, and we show the underlying mechanism. We then utilize the mechanism by demonstrating transient CXCR4 upregulation in a clinically translatable LTRC population to specifically increase GM LTRC engraftment when transplanted i.v. and to remarkably enhance engraftment of GM LTRC when transplanted i.b.m.

There have been numerous studies aimed at using i.b.m. transplantation as a more effective means of HSPC delivery. Many animal studies suggest that i.b.m. delivery results in higher early engraftment and homing than does i.v. delivery (42–44). However, the impact on long-term engraftment is debated, with some studies showing higher engraftment with i.b.m. delivery (45–47) and others showing no significant improvement (4, 16, 45, 48–50). I.b.m. transplantation has also been shown as an efficient means of studying limited cell number or difficult-to-engraft populations in animals, suggesting that i.b.m. transplantation corresponds to higher cell homing and retention (44, 51–55). Clinical studies suggest that i.b.m. delivery may lessen graft-versus-host disease and bacterial complications (56), as well as lessen the time to neutrophil and platelet engraftment (11–13, 57). Comparing the patients in these studies to historic controls suggests that the overall transplant outcome is not significantly altered. To date, the only clinical study using i.b.m. transplantation in a gene therapy context showed that i.b.m. delivery was safe and yielded long-term stable engraftment of GM cells (4). However, this clinical study did not have an i.v. comparative group, and the corresponding animal study showed superiority of i.b.m. at both 2 and 4 months that was lost by 7 months (4), similar to our data herein.

Reliability of HSPC delivery in the BM space when injected i.b.m. has been questioned as a source of disparate results. Several studies have looked at cell retention in the BM following i.b.m. delivery and have shown that more than 90% of injected cells leave the BM immediately following injection and are found at other sites (14–16, 58). Using a porcine model, Pantin et al. show that this cell loss is due to injection rate and i.b.m. pressure and can be mitigated by modification of injection method (14). Shi et al. support this data showing the importance of BM retention on long-term engraftment using a collagen gel matrix to enhance cell retention (59). Our homing and bioluminescence data from mice, however, suggest that when HSPC are delivered via a small-volume i.b.m. injection, the majority of HSPC are retained in the injected space, as opposed to immediately entering circulation.

We believe our study identifies the mechanism that explains disparate short- and long-term engraftment data reported on i.b.m. transplantation. While CXCR4 is a well-established homing receptor on HSPC, we show that higher CXCR4 expression on HPC gives HPC a homing and engraftment advantage over lower CXCR4-expressing HSC following i.b.m. delivery. Removing CXCR4^hi^ HPC and transplanting an HSC-enriched population removes competition and results in improved long-term engraftment following direct BM transplantation. In an effort to minimize LV vector usage and reduce associated costs, transplantation of an HSC-enriched CD34^+^CD38^–^ or CD34^+^CD38^–^CD90^+^ population is coming into favor (7, 34). As this becomes the standard of care, direct BM transplantation may be a relevant clinical option to reduce homing losses and achieve improved transplant success.

Alternatively, we show that transient protein delivery of CXCR4 in the LV particle can be used to improve homing and engraftment specifically of GM HSPC, even when delivered i.v., without altering the homing and engraftment of untransduced HSPC. This method has been used previously to fluorescently label HIV virions to track viral entry into cells (60, 61). We have utilized published structural functional analysis to remove all cytotoxic domains from VPR while retaining gag binding. Using protein delivery allows for the cells to have an advantage during the critical homing time period but does not risk causing future immunodeficiency resulting from permanent CXCR4 upregulation. Additionally, by fusing CXCR4 to VPR and carrying it as a protein rather than as a LV gene, we avoided the issues of transgene size and double transduction by both a transgene-carrying LV and a nonintegrating CXCR4-encoding LV that have impaired previous efforts.

We also show that CXCR4 delivered via VLP can increase CXCR4 cell surface expression, and hence VLP^CXCR4^ (Figure 5B) can be used in conjunction with gene editing approaches and even UCB transplants, expanding the use of this approach beyond LV vectors. Finally, by either using CXCR4 or substituting CXCR4 with other homing molecules, this VPR protein delivery method could be used to target other cell-based therapies such as chimeric antigen receptor T (CAR-T) and CAR-NK cells to their specific homing sites.

## Methods

### Human CD34^+^ HSPC isolation, culture, and transduction.

UCB and granulocyte CSF (G-CSF) MPB were obtained using CCHMC Institutional Review Board–approved protocols, and CD34^+^ HSPC were isolated by magnetic selection with the human Indirect CD34 Microbead kit (Miltenyi Biotec, 130-046-701). CD34^+^ purity was > 95% as confirmed by flow cytometry. CD34^+^CD38^–^ cells were isolated from G-CSF MPB by magnetic selection with human CD34^+^CD38^–^ Isolation Kit (Miltenyi Biotec, 130-114-822) and cryopreserved. Thawed CD34^+^ or CD34^+^CD38^–^ cells were cultured in X-VIVO 10 (Lonza, BE02-055Q) or SCGM (CellGenix, 20806-0500) supplemented with 2% human serum albumin, 100 ng/mL TPO, 300 ng/mL SCF, and 300 ng/mL FLT3-L (all cytokines purchased from Peprotech) at a cell density of 2 × 10^6^ to 5 × 10^6^ CD34^+^ cells/mL. Media was supplemented with Birb 796 (600nM) (Selleckchem) throughout culture and PGE2 (10 μM) (Cayman) at plating, transduction, and 1 hour before harvest. Cells were transduced with a LV vector in culture for 36–42 hours where indicated. CD34^+^ HSPC were transduced at a final concentration of 5 × 10^7^ to 1 × 10^8^ IU/mL. Following culture, CD34^+^ HSPC were harvested and washed with PBS, resuspended in PBS, and used in in vivo or in vitro experiments, as described below.

### Mouse xenograft model.

Six- to 14-week-old male and female NSG mice (The Jackson Laboratory) were used in UCB- and MPB-derived HSPC transplant experiments. Mice were irradiated with 280 cGy prior to transplant. For homing experiments, each mouse received 3 × 10^6^ CD34^+^ cells, and 16–22 hours after injection, the mice were sacrificed and BM from the femurs was analyzed for human cell content. For long-term engraftment experiments, mice received limiting dilution CD34^+^ or CD34^+^CD38^–^ HSPC doses. I.v. injections were done by injecting HSPC in 200–250 μL via tail vein and i.b.m. injections were done by injecting HSPC in 10 μL into the femur.

### Lentiviral vectors.

All LV vectors were packaged using the gag-Pol, Rev, and VSV-G envelope plasmids. All vectors were under the control of the MNDU3 promoter. The GFP LV construct was pRRL.SIN.cPPT.MNDU3.eGFP.WPRE. The BFP LV construct was designed by replacing the GFP cDNA in pRRL.SIN.cPPT.MNDU3.eGFP.WPRE. with mTag2BFP. The BFP-CXCR4 LV was pRRL.SIN.cPPT.MNDU3.mTag2BFP-CXCR4.WPRE. All LV vectors were packaged in HEK 293T (ATCC) cells, and vector titers were determined in the murine erythroleukemia (MEL) (ATCC) cells as previously described (62–64).

### Protein delivery of CXCR4 in the LV vector.

A VPR-CXCR4 fusion was created by fusing CXCR4 cDNA to a synthesized truncated and mutated VPR sequence. A 78 aa truncated VPR sequence was designed, lacking the 18 aa from the carboxy terminus to remove a major nuclear localization signal and the portion that is critical for VPR-mediated cell cycle arrest (35, 39, 41). Three point mutations were introduced: a Q65R mutation that abrogates its binding to DCAF and is the primary event that triggers a change in the cellular proteome of viral particle–delivered VPR, including depletion of cell cycle regulatory proteins and proteins involved in DDR (36), and R77Q and W54R point mutations, which further prevent its cytotoxic activities and reduce its stability. These are mutations were seen in long-term nonprogressors with HIV infection (37, 40, 65). This VPR^MT^ that retains its ability to bind gag (the LV capsid protein) but lacks VPR-associated cytotoxicity was then fused to CXCR4 using the HIV-1 PCS (38) so that CXCR can be cleaved from VPR^MT^ by the protease present in the LV particle. This fusion plasmid was used along with the other packaging plasmids to package a GFP-encoding LV vector, using the GFP LV construct listed in LV vectors.

### Bioluminescent imaging.

Mice received an i.p. injection of 150 mg/kg body weight D-luciferin (Xenogen, XR-1001) 15 minutes before being anesthetized with 3% isoflurane. Anesthetized animals were imaged with the Perkin Elmer In Vivo Imaging System (IVIS) and then allowed to recover fully from anesthesia.

### Secondary transplants.

BM was harvested from primary transplanted NSG mice at 24 weeks after transplant. BM underwent magnetic mouse CD45 antibody depletion using Biotin Rat anti–mouse CD45 (BD Biosciences, 553078) and Streptavidin Particles Plus (BD Biosciences, 557812) and was transplanted 1:1 i.v. into irradiated (280 cGy) NSG mice. Secondary mice were analyzed at 12 weeks after transplant.

### CXCR4 adhesion assay.

CXCR4 ligand affinity was measured by adhesion assay (66). Tissue culture–treated flasks were coated with 2.5 μg/mL CXCL12 (Peprotech, 300-28A) overnight at 4°C, washed with PBS, and then 2 × 10^6^ CD34^+^/mL were plated on the CXCL12-coated plate and incubated at 37°C for 2 hours. Following the 2-hour incubation, nonadherent HSPC were removed. Adherent cells were harvested with rigorous flushing with PBS.

### AMD3100.

CD34^+^ HSPC were resuspended in media containing 100 μg/mL AMD3100 (MilliporeSigma, A5602) for 37°C for 15 minutes. The cells were washed with PBS and transplanted into NSG mice for homing experiments.

### BIO5192.

CD34^+^ HSPC were resuspended in PBS containing 1 μM BIO5192 (Tocris, 5051) and incubated on ice for 20 minutes. The cells were washed with PBS and transplanted into NSG mice for homing experiments.

### Flow cytometry.

For HSC analysis, CD34^+^ cells were stained with the following antibodies: PE-Cy7 Mouse anti–human CD34 (s, 560710) or CD34 APC (BD Biosciences, 555824), anti-human CD38 APC eFluor 780 (eBioscience, 47-0389-42), Alexa Fluor 700 anti–human CD90 (BioLegend, 328120), and anti–human CD45RA APC (eBioscience, 17-0458-42). For VLA4 staining, PE mouse anti–human CD49d (BD Biosciences, 555503) was used. For CXCR4 staining, PE-Cy7 anti–human CD184 (CXCR4) (BioLegend, 306514) was used. For cell cycle staining, cells were stained with cell surface antibodies, fixed using BD Fix and Perm (BD Biosciences, 554714), and then stained with Hoescht-33412 (BD Biosciences). For annexin V and 7AAD staining, PE annexin V Apoptosis Detection Kit with 7-AAD was used (BioLegend, 640934). γH2AX was stained with PE γH2AX (BioLegend, 613411).

At 12 weeks following transplant, BM was harvested via aspirate of the femur for in vivo engraftment analysis. For animals that received i.b.m. injections into 1 femur, the noninjected alternate femur was aspirated. For 24 week and homing analysis, mice were sacrificed, and BM was harvested from both the rear and forelimbs. Cells were stained fresh with the following antibodies: PerCP anti–human CD45 (BioLegend, 304026), anti–human CD33 PE-Cy7 (eBioscience, 25-0338-42), APC-Cy7 mouse anti–human CD19 (BD Biosciences, 557791), anti–human CD3 PE (BD Biosciences, 555340), and APC mouse anti–human CD34 (BD Biosciences, 555824). RBCs were lysed after staining using red blood cell lysis buffer.

Stromal cells were analyzed by staining cells with: Biotin anti–mouse CD51 (BioLegend, 104104), FITC anti–mouse CD45 (BioLegend, 368508), and Alexa Fluor 700 anti–mouse lineage cocktail (BioLegend, 133313).

### Statistics.

All statistical analyses were performed in GraphPad Prism. In all experiments, groups of 2 were compared by Mann-Whitney *U* test (1-sided), and those greater than 2 were analyzed by ANOVA with correction of multiple comparisons or with 2-way ANOVA. All data are plotted as mean ± SEM, except where indicated. *P* < 0.05 was considered significant, with degree of significance indicated. *P* values trending toward significance and pertinent nonsignificant are indicated on the graphs.

### Study approval.

All human samples were collected under IRB-approved protocols at CCHMC. All donors gave their written informed consent prior to inclusion in the study. All animal work was performed under CCHMC IACUC–approved protocols.

## Author contributions

PM originated the study concept. PM and SF designed experiments and prepared the manuscript. SF carried out experiments and performed data analysis. AS assisted in experiments related to NSG transplants and HSPC culture. DMP assisted in vector design and cloning. DS assisted in vector production and titration. JB assisted with all animal transplant and procedures.

## Supplementary Material

Supplemental data

## Version 1 05/09/2022

Electronic publication
