# Supplementary material for: Differential CXCR4 expression on hematopoietic progenitor cells versus stem cells directs homing and engraftment
Source: JCI Insight. 2022 May 9;7(9):e151847. doi: 10.1172/jci.insight.151847 (PMC9090236; doi:10.1172/jci.insight.151847)
Supplement: Supplemental data [file jciinsight-7-151847-s175.pdf]

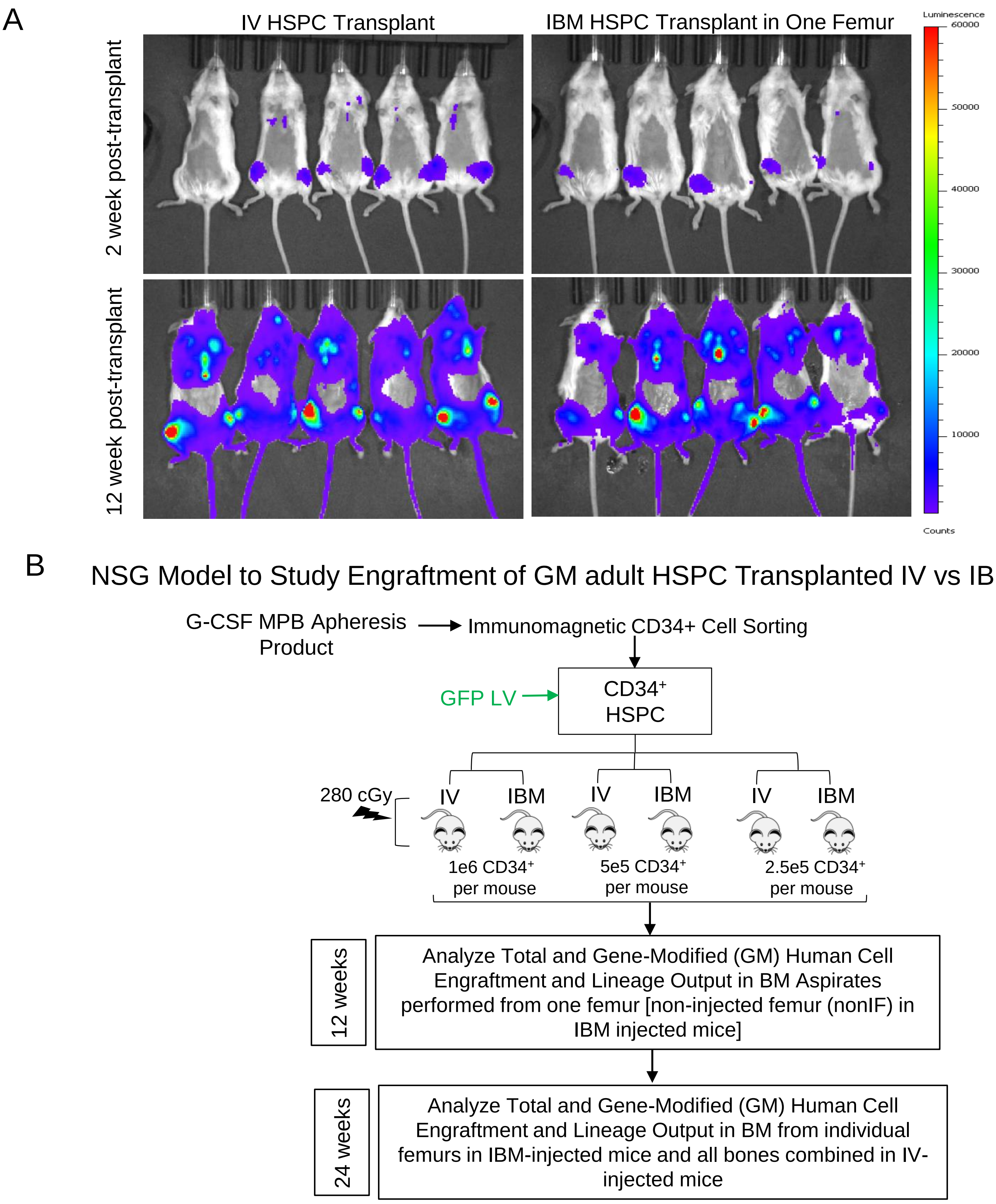

Figure S1. Comparison of intra-bone marrow (IBM) and intravenous (IV) transplant of hematopoietic stem and progenitor cells (HSPC). (A) Determining accuracy of IBM transplant. CD34+ hematopoietic stem and progenitor cells (HSPC) were isolated from umbilical cord blood and transduced with a luciferase-encoding lentivirus vector; 250,000 HSPC were either transplanted IV into the tail vein (left panels) or IBM into the right femur (right panels). Mice were imaged on the Perkin Elmer IVIS after D-luciferin injection. The earliest detection of adequate luciferase signal occurred at 2 weeks (top panels), showing localization of luciferase signal in bone marrow. By 12 weeks (bottom panels), there was widespread engraftment of luciferase-marked HSPC. (B) NSG model to study engraftment of GM adult HSPC derived from G-CSF mobilized peripheral blood were transplanted limiting dilution CD34+ cell doses after transduction of the cells with a GFP-encoding LV vector either IV or IBM. Indicated cell doses were injected after NSG mice received 280cGy irradiation. At 12 weeks after transplant, short-term engraftment was analyzed via bone marrow aspirate from one femur (the non-injected femur in IBM mice). At 24 weeks after transplant, mice were sacrificed and analyzed for long-term engraftment.

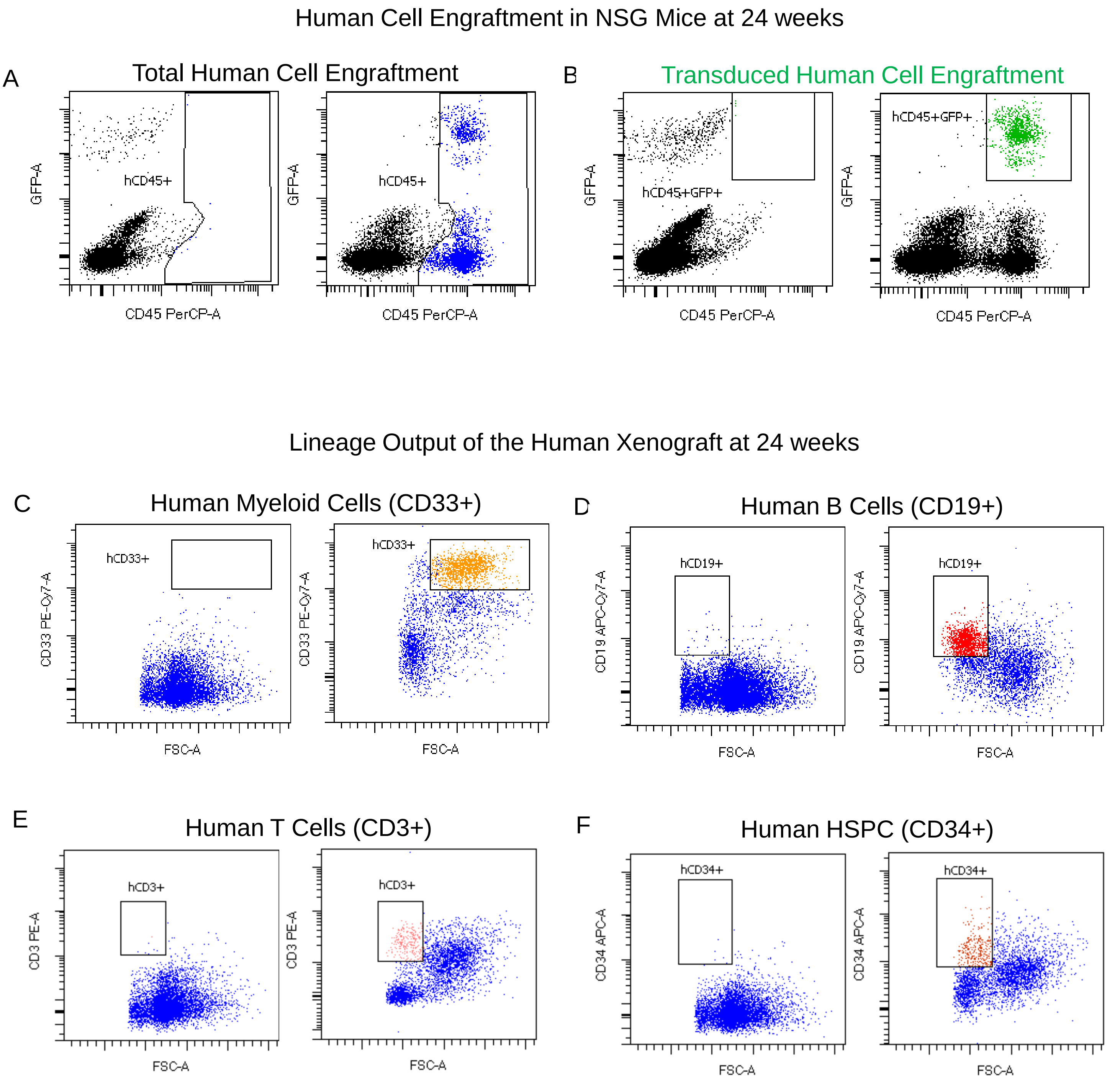

Figure S2. Representative flow cytometry dot plots showing human engraftment and lineage output at 24 weeks after transplant. (A) Total human cell engraftment was assessed by labeling bone marrow from the xenografted NSG mice with hCD45 Ab and gating hCD45+ cells as shown in the right panel with corresponding unstained control in the left panel. (B) Transduced human cell engraftment was assessed by gating on hCD45+ GFP+ cells, shown in the right panel with the unstained controls in the left panel. (C-E) Human cell lineages, myeloid cells, B cells, T cells and CD34+ cells were quantified by labeling the xenograft bone marrow with hCD33 Ab, hCD19 Ab, hCD3 Ab and hCD34 Ab, respectively, with the labelled populations in the right panels and respective controls in the left panels.

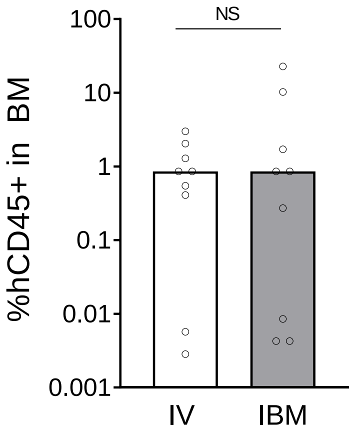

Figure S3. Secondary transplants show no difference in long term engraftment with IV or IBM delivery. Pooled bone marrow from femurs, tibias, and iliac crests from primary mice at 24 weeks was transplanted into sub-lethally irradiated secondary mice. Secondary mouse bone marrow was analyzed for human cell engraftment at 12 weeks. Bars represent median engraftment. Each symbol represents an individual mouse. N=9 secondary animals per group; Primary mice were transplanted from one MPB donor. Transduced cell engraftment is not shown as the overall percentage of transduced cells was very low and therefore not reliable. Symbols represent individual secondary mice; statistical analysis performed by Mann-Whitney test.

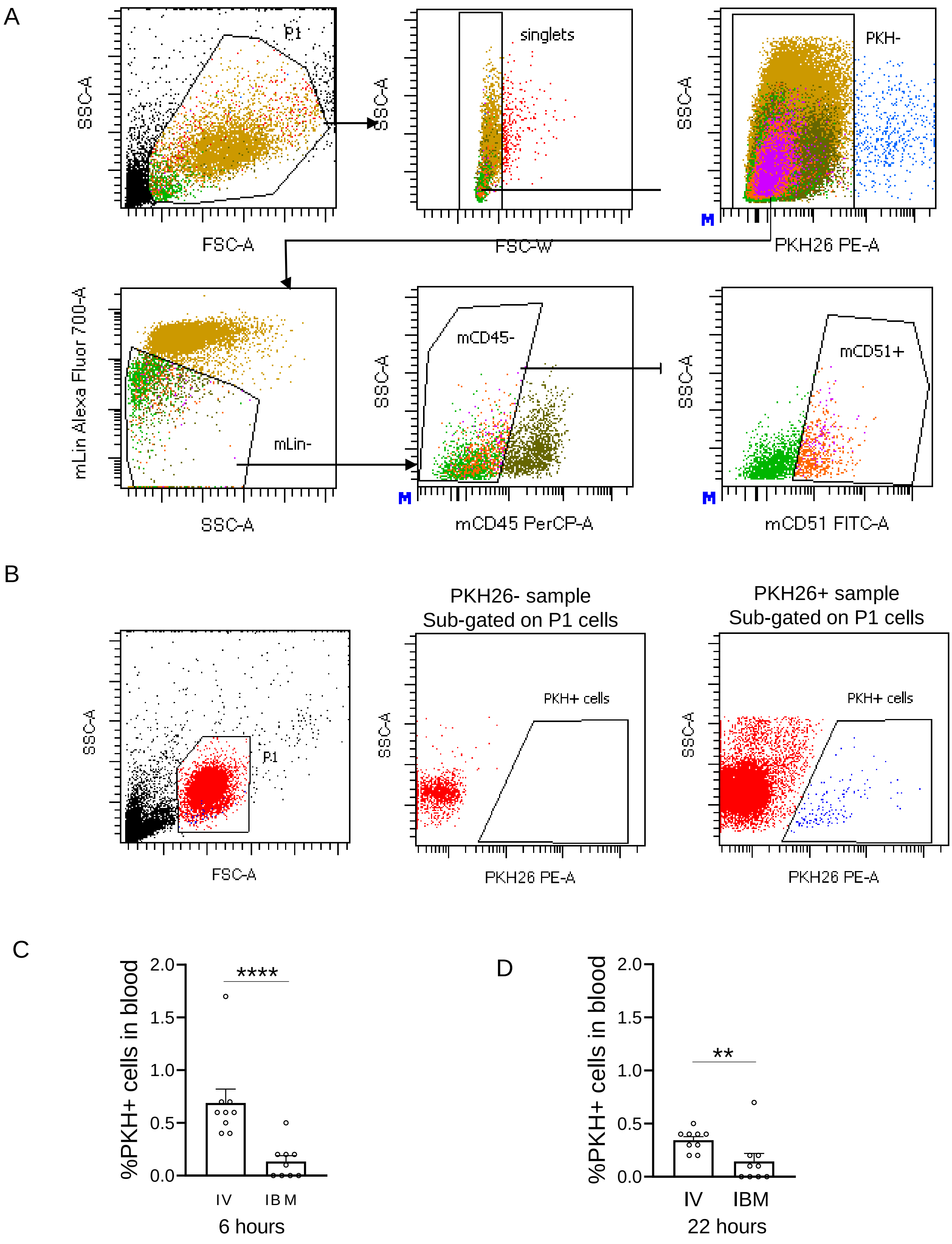

Figure S4. Analysis of blood and stromal cells following IV and IBM injection. (A) Representative flow cytometry gating for stromal cells. Bone marrow was harvested from mice 22 hours post-transplant of PHK26-labelled human CD34+ cells by IV or IBM injection. Cells were stained for stromal markers and analyzed as indicated. (B) NSG mice were transplanted with 3e6 CD34+ cells pre-labelled with PKH26 via IV or IBM injection. Whole blood was subjected to red blood cell lysis and then acquired by flow cytometry for PKH+ cells. Representative flow cytometry plots showing gating for PKH26+ (PKH+) cells from whole blood. (C,D) Mice were bled at 6 hours (C) and 22 hours (D) post-transplantation and blood analysis for PKH+ cells is shown after IV or IBM injection. N=9; statistical analysis was performed using Mann-Whitney test.

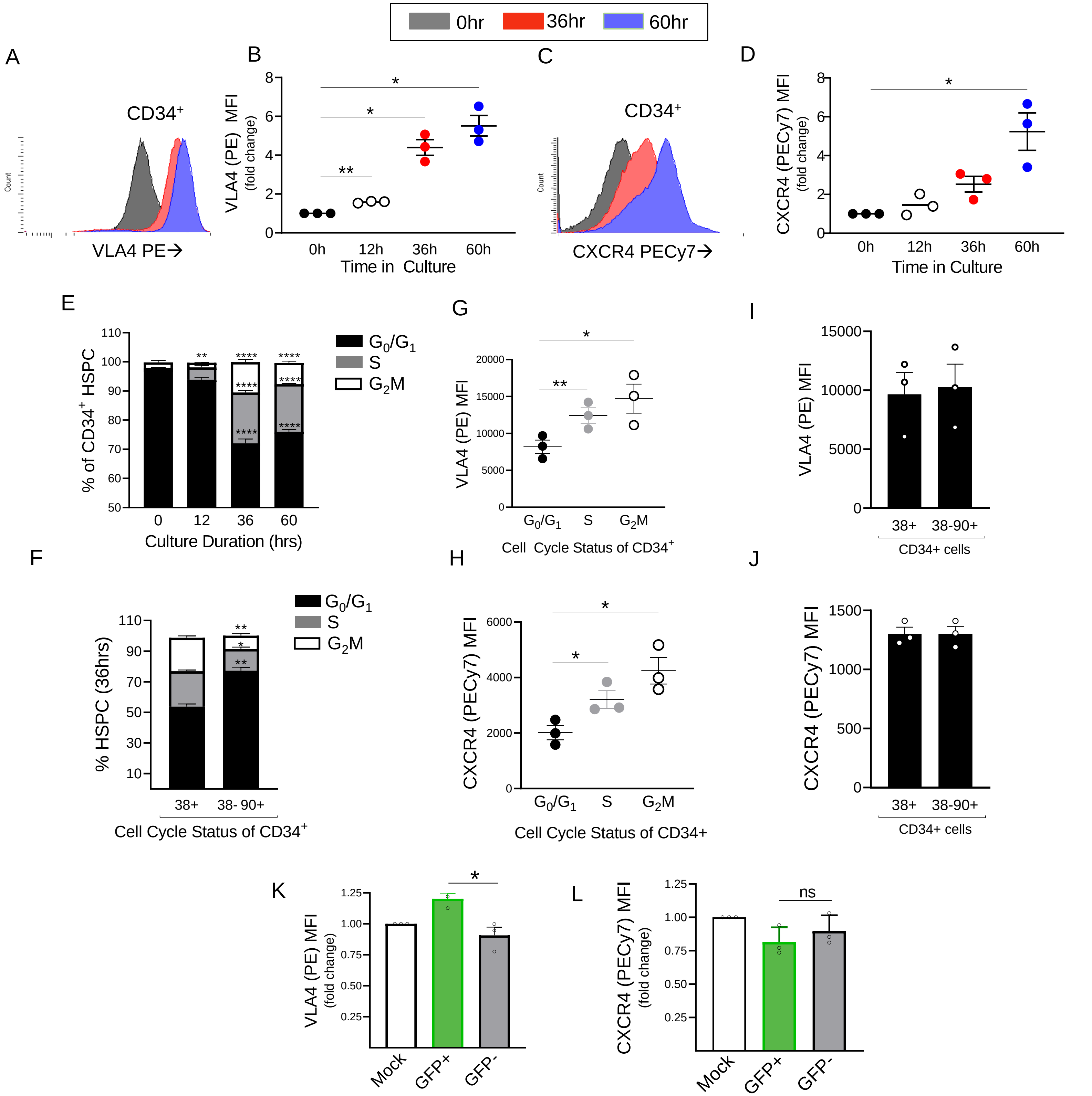

Figure S5. Effect of ex vivo culture, cell cycle, and transduction on homing receptor expression on HSPC. (A-D) Cell surface expression of VLA4 (A, B) and CXCR4 (C,D) on CD34+ HSPC increases with increasing time in culture. Each symbol represents a unique MPB donor. N=3. Statistical comparisons are performed using ANOVA. (E-H) Cell cycle status of CD34+ HSPC in culture and its effect on CXCR4 and VLA4 homing receptor expression. (E,F) Cell cycle analysis of HSPC: (E) CD34+ HSPC are largely quiescent when harvested but increasing numbers of HSPC enter cell cycle with increasing time in culture. (F) Of the HSPC population, significantly higher numbers of the CD34+CD38+ HPC are in cycle, as compared to relatively low numbers of cycling HSC (CD34+38-90+). N=3; statistical analysis performed using ANOVA. (G,H) Both VLA4 and CXCR4 expression is increased on cycling HSPC. Each symbol represents a unique MPB donor; N=3; statistical comparisons performed using ANOVA. (I, J) Homing receptor expression is cell cycle dependent. Non-cycling (G0 phase) CD34+38+ HPC and CD34+38- HSC enriched cells express similar levels of homing receptor expression. (K, L) Effect of transduction on homing receptor expression. CXCR4 expression in transduced and untransduced cells is similar, but VLA4 expression is significantly higher on transduced HSPC. N=3 unique biological replicates; statistical analysis performed by ANOVA between populations. * p<0.05, **p<0.01, *** p<0.001 , **** p<0.0001

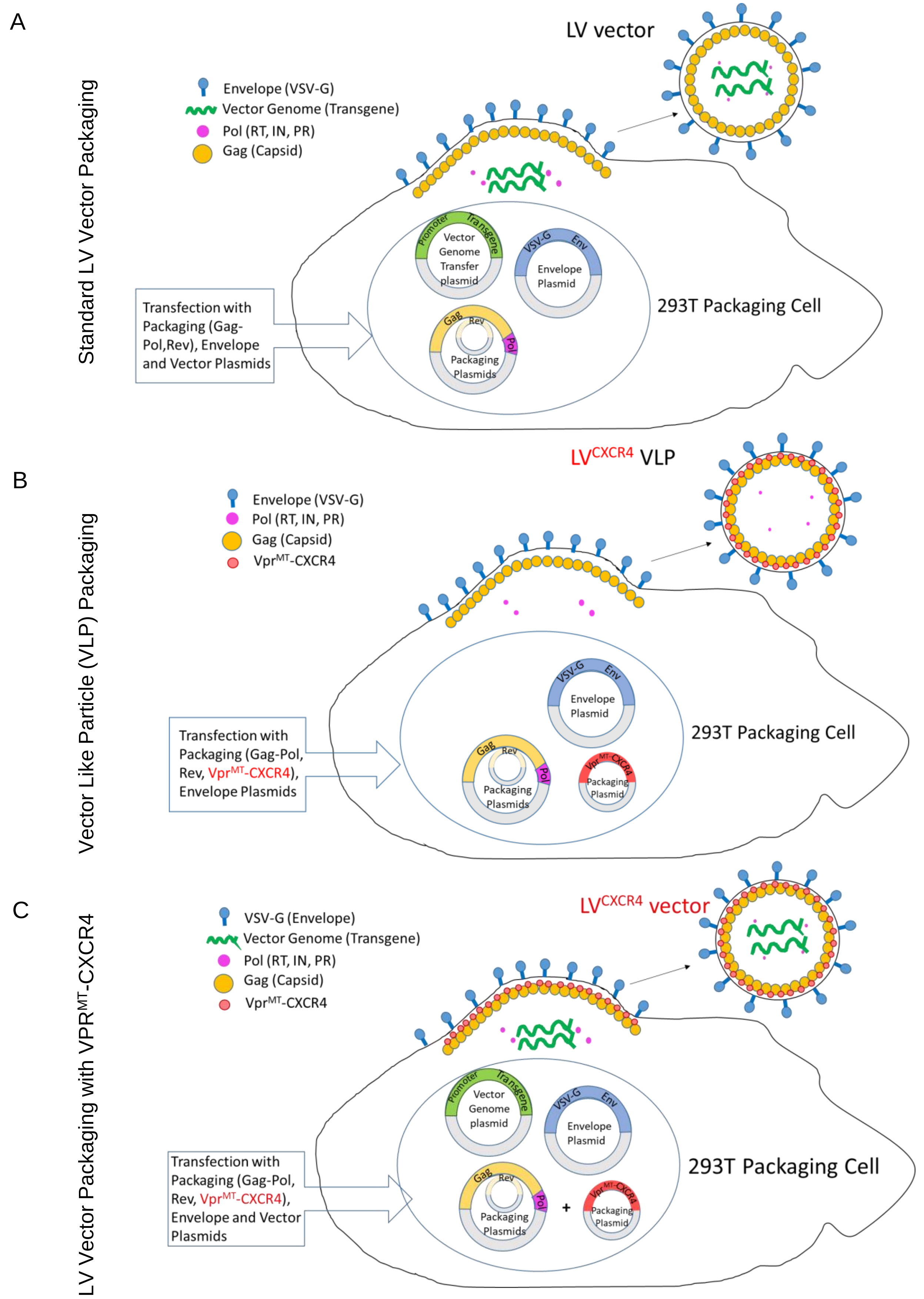

Figure S6. (A) Schematic of packaging of a lentiviral (LV) vector using the 'standard packaging plasmids': (i) Gag-Pol [gag forms the capsid protein, pol generates the reverse transcriptase (RT), integrase (IN) and protease (PR)] and (ii) Rev (facilitates full length vector genome mRNA export) and (iii) the envelope plasmid (VSV-G). The transgene/vector genome plasmid (that carries the marker/therapeutic transgene) carries the ψ sequences allowing the transgene sequences to be packaged in the virion. (B). LV^{CXCR4} vector like particles (VLP) packaged with the standard packaging plasmids and the additional Vpr^{MT}-CXCR4 packaging plasmid (LV^{CXCR4}) but without the vector genomic/transgene plasmid to generate empty LV particles is shown. (C) LV vector particles packaged with the transgene/vector genome plasmid, the standard packaging plasmids and the additional Vpr^{MT}-CXCR4 packaging plasmid (LV^{CXCR4}) is shown.

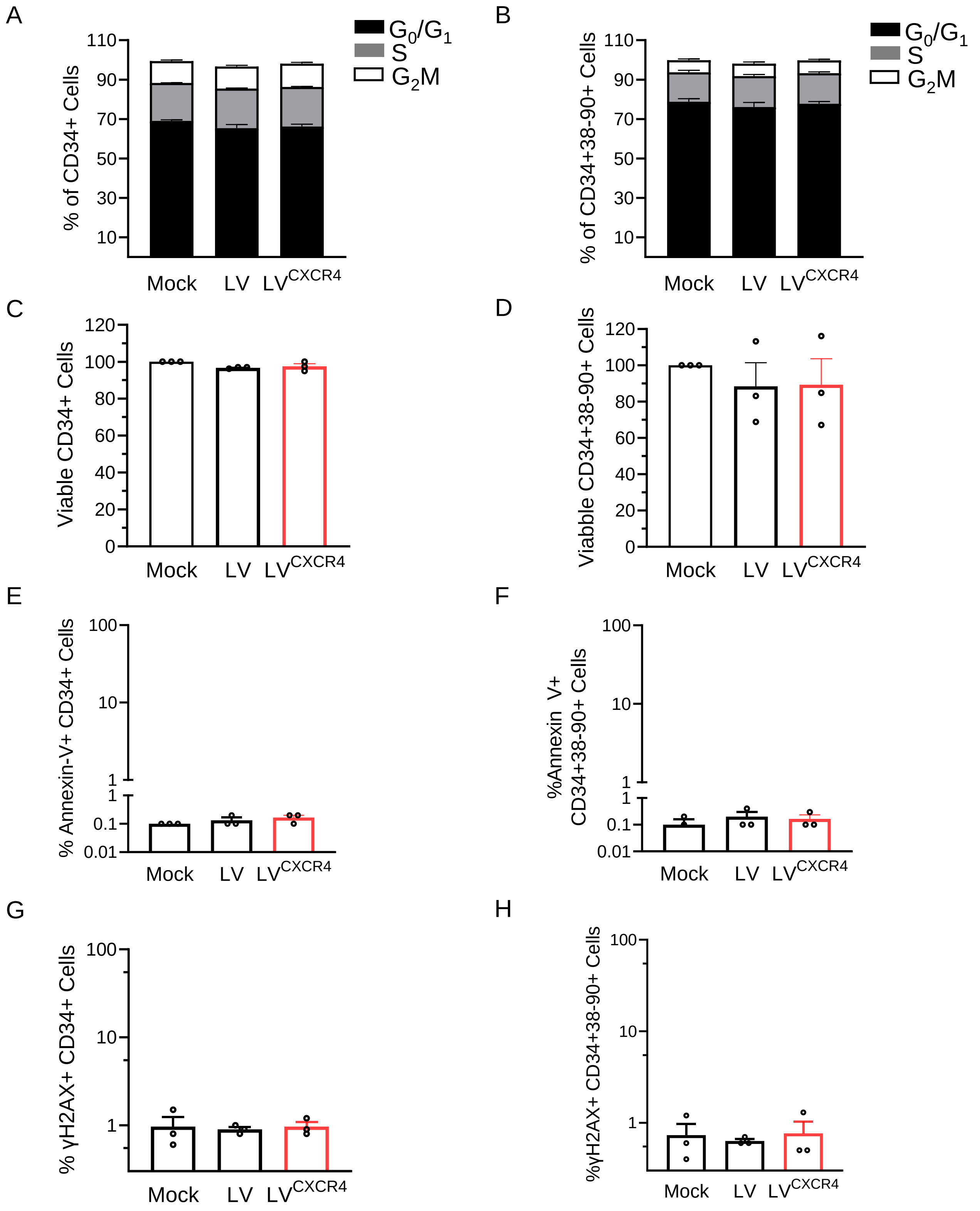

Figure S7. Protein delivery of CXCR4 within a LV fused to VPR^{MT} lacks the toxicity in CD34+ HSPC that is normally associated with wild type VPR protein in T lymphocytes. CD34+ cells were either transduced with a GFP LV vector or a GFP LV^{CXCR4} vector. Gene transfer was comparable between groups for all donors. (A, B). Cell cycle status was determined in CD34+ HSPC and CD34+CD38-CD90+ HSC populations 72 hours-post transduction. Earlier time-points did not have sufficient CD34+cD38-CD90+ cells in G_2M phase for a meaningful comparison. Statistical analysis was performed using 2-way ANOVA, with no significant differences between Mock, LV and LV^{CXCR4}. (C,D). Viable cell counts of CD34+ HSPC and CD34+CD38-CD90+ HSC were determined 48 hours post-transduction. Cell counts were normalized to untransduced (mock) control cell counts from each unique donor. Symbols represent unique MPB donors; N=3. (E,F). Apoptosis was measured in CD34+ HSPC and CD34+CD38-CD90+ HSC populations 48 hours post-transduction via Annexin V staining. Symbols represent unique MPB donors; N=3. (G, H). DNA damage response was assessed by **γ**H2AX+ cells in both CD34+ and CD34+CD38-CD90+ HSC populations 48 hours post-transduction. Symbols represent unique MPB donors; N=3. For panels C-H, Statistical analysis was performed using ANOVA with no significant difference between Mock, LV and LV^{CXCR4}.

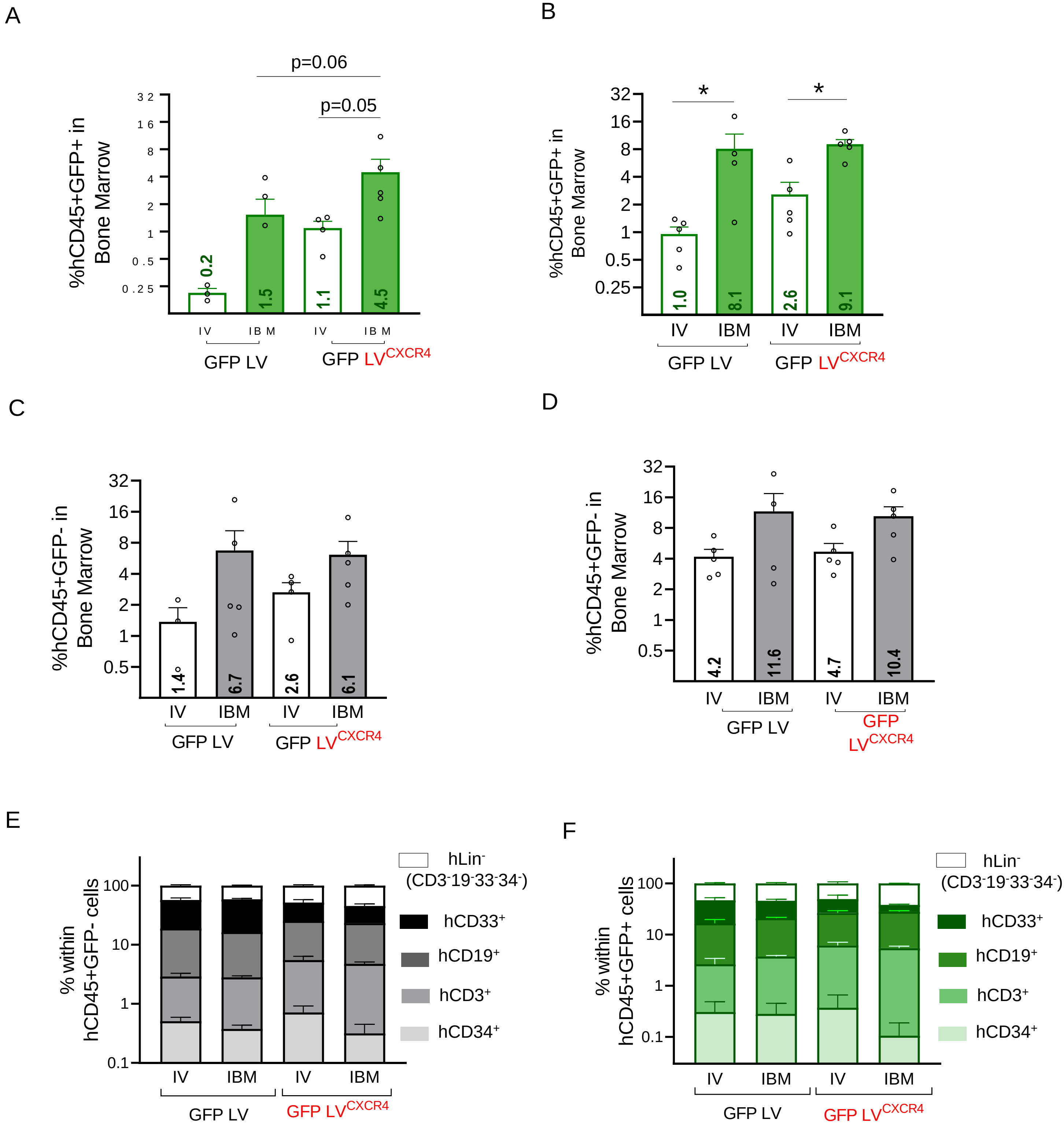

Figure S8. Protein delivery of CXCR4 enhances long-term multilineage engraftment of CD34+CD38- HSC-enriched cells when transplanted IV or IBM. (A-D) Irradiated NSG mice were transplanted 0.25e5 (A, C) or 0.5e5 (B, D) CD34+CD38- HSC-enriched cells that were transduced with GFP LV or GFP LV^{CXCR4} (gene correction was 60% with GFP LV and 52% with GFP LV^{CXCR4}) by either IV or via IBM injection at the indicated cell doses. Human cell engraftment was analyzed by determining the percentage of gene-modified/transduced human CD45+ (hCD45+GFP+) (A,B) cells and the percentage of non-transduced (hCD45+GFP-) (C,D) cells in bone 24 weeks post-transplant. Symbols represent individual mice; N=3-5 mice per cell dose per group; statistical analysis performed using ANOVA. (E, F) Lineage output of the human xenograft. Bone marrow was stained antibodies specific for T cell lineage (hCD3), B cell lineage (hCD19), myeloid lineage (hCD33) and HSPC (hCD34) to determine the lineage output of the transduced and untransduced HSC-enriched population 24 weeks post-transplant. Statistical analysis was performed using 2-way ANOVA. * $p<0.05$, ** $p<0.01$.
